# Antiproliferative Phenothiazine Hybrids as Novel Apoptosis Inducers against MCF-7 Breast Cancer

**DOI:** 10.3390/molecules23061288

**Published:** 2018-05-28

**Authors:** Jun-Xia Zhang, Jiao-Mei Guo, Ting-Ting Zhang, Hong-Jun Lin, Nai-Song Qi, Zhen-Guo Li, Ji-Chun Zhou, Zhen-Zhong Zhang

**Affiliations:** 1 School of Pharmaceutical science, Zhengzhou University, Zhengzhou 450001, China; zjx312@163.com; 2 Department of Pharmacology, Henan Provincial Institute of Food and Drug Control, Zhengzhou 450008, China; ssjsel745288@163.com (J.-M.G.); 18637123006@163.com (T.-T.Z.); 13393723919@163.com (H.-J.L.); 18538293031@163.com (N.-S.Q.); 13298181239@163.com (Z.-G.L.); 18838969948@163.com (J.-C.Z.); 3 Key Laboratory of Targeting Therapy and Diagnosis for Critical Diseases, Henan Province, Zhengzhou 450001, China

**Keywords:** phenothiazine-1,2,3-triazole, MCF-7, apoptosis, DR5

## Abstract

We designed a series of novel phenothiazine-1,2,3-triazole hybrids by the molecular hybridization strategy and evaluated their antiproliferative activity against three cancer cell lines (MDA-MB-231, MDA-MB-468 and MCF-7). For the structure-activity relationships, the importance of 1,2,3-triazole and substituents on phenyl ring was explored. Among these phenothiazine-1,2,3-triazole hybrids, compound **9f** showed the most potent inhibitory effect against MCF-7 cells, with an IC_50_ value of 0.8 μM. Importantly, compound **9f** could induce apoptosis against MCF-7 cells by regulating apoptosis-related proteins (Bcl-2, Bax, Bad, Parp, and DR5). These potent phenothiazine-1,2,3-triazole hybrids as novel apoptosis inducers might be used as antitumor agents in the future.

## 1. Introduction

According to the cancer epidemiology data collected in the cancer statistics 2017, breast cancer accounts for about 14.4% of cancer related death and 29.6% of new cases in female, and which is the top type of female new cancer cases and the second cause of female cancer related death worldwide after lung cancer [1]. Its poor overall prognosis reflects not only its inherent aggressiveness but also the lack of targeted therapies [2]. Therefore, it is necessary to develop the potent antitumor agents to treat breast cancer.

**Figure 1:**
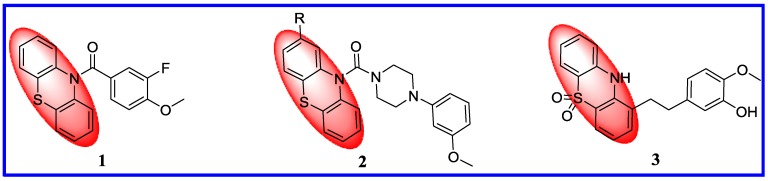
Phenothiazine derivatives as novel anticancer agents.

Phenothiazine is a useful scaffold in antitumor drug design [3,4,5]. Recently, many phenothiazine derivatives were designed as novel anticancer agents. For example, phenothiazine derivative **1** (Figure 1) proved to be a microtubule-interacting agent with great cell growth inhibition profile (GI_50_ in the nanomolar range on human melanoma-adenocarcinoma MDA-MB-435 cells) [6]. Phenothiazine derivative **2** could arrest tumor cell cycle in the G2/M phase [7]. The 3-hydroxy-4-methoxyphenylethyl derivative **3** was a potent in vitro tubulin polymerization inhibitor but with weak cytotoxicity toward the human colon adenocarcinoma HT29 cell line, in the micromolar range [8].

**Figure 2:**
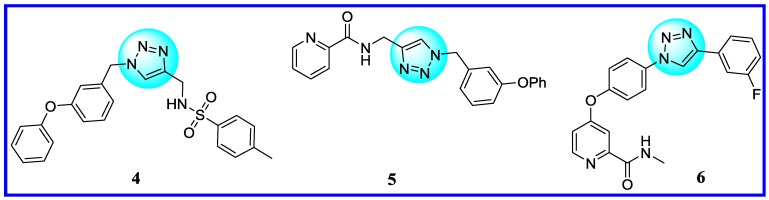
1,2,3-Triazole derivatives as novel antitumor agents.

Triazole derivatives display an ample spectrum of pharmacological activities and are widely employed as pharmaceuticals and agrochemicals [9]. They have been regarded as important skeletons in terms of biological activity and some of them have also shown significant anticancer activity [10]. Benzenesulfonamide **4** (Figure 2) showed an IC_50_ value of 4.08 μM against PC-3 cells and the potent tubulin depolymerization activity in vitro with an IC_50_ value of 2.41 μM. Arylamide-1,2,3-triazole **5** exhibited potent antitumor in vitro against MCF-7 cancer cells (IC_50_ = 46 nM) [11]. Picolinamide-1,2,3-triazole **6** could induce apoptosis and colony formation at a low micromolar concentration against HT-29 cancer cells [12].

**Figure 3:**
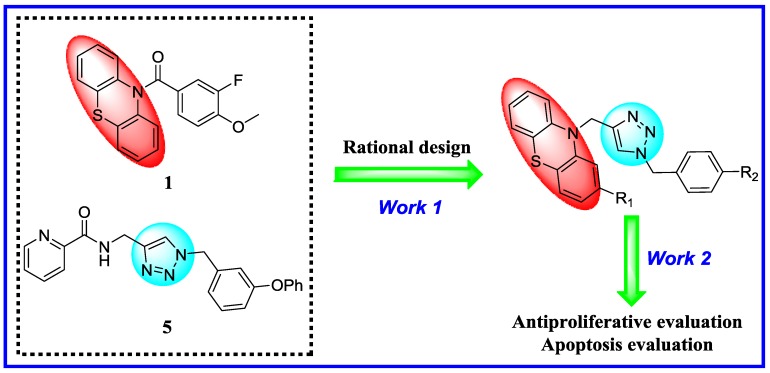
Rational design of target compounds as novel antitumor agents.

Based on the results of 1,2,3-triazole and phenothiazine as antitumor skeletons, we envisioned that a phenothiazine ring linked a 1,2,3-triazole unit would serve as a novel and potent anticancer agents. In this study, the synthesis and antiprolifervative evaluation of phenothiazine-1,2,3-triazole analogues, as well as the inducing apoptosis properties for the most active compound were explored (Figure 3). 

## 2. Results and Discussion

### 2.1. Chemistry

**Figure 4:**
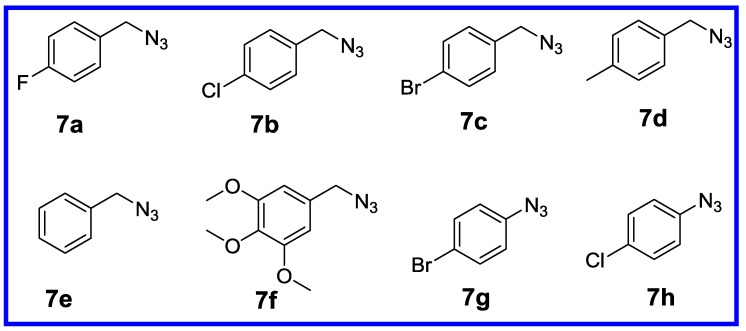
Azide derivatives **7a**–**7h** used in this study.

Azide derivatives **7a**–**7h** used in this paper were purchased from Beijing Research Biotechnology Co., Ltd (Beijing, China) (Figure 4). Commercially available phenothiazine was treated with propargyl bromide to provide **8a**–**8b** in the presence of potassium carbonate.

**Scheme 1:**
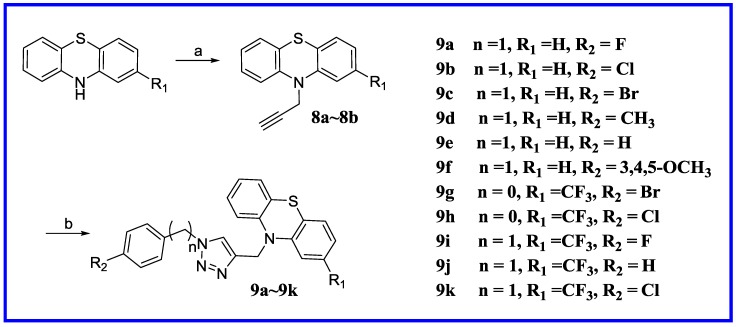
Synthesis of phenothiazine-1,2,3-triazole hybrids **9a**–**9k**. Reagents and conditions: (**a**) K_2_CO_3_, dichloromethane, propargyl bromide, reflux; (**b**) CuSO_4_.5H_2_O, sodium ascorbate, azide derivatives **7a**–**7h**, THF:H_2_O (1:1), rt.

Azide–alkyne 1,3-dipolar cycloaddition reaction popularly known as the “click chemistry” serves as the highly dependable tool for facile construction of antiproliferative agents [13,14]. These novel phenothiazine hybrids **9a**–**9k** were obtained from alkyne intermediate **8a**–**8b** and azide derivatives **7a**–**7h** using sodium ascorbate and copper sulfate system in THF/H_2_O (Scheme 1). (The spectra of compounds **9a**–**9k** can be seen in the supplementary materials.)

### 2.2. Antiproliferative Activity and SARs

All the synthesized and target phenothiazine-1,2,3-triazole hybrids **9a**–**9k** were evaluated for their antitumor activity in vitro against three breast cancer cell lines (MDA-MB-468, MDA-MB-231 and MCF-7) using a reported MTT assay [15]. In our antiproliferative experiments, 5-fluorouracil (5-Fu) was used as a control [16,17]. Table 1 describes the antiproliferative activity results of phenothiazine-1,2,3-triazole hybrids **9a**–**9k** and phenothiazine derivatives **8a**–**8b**.

To explore the inhibitory effect of 1,2,3-triazole unit, phenothiazines **8a**–**8b** and **9a**–**9k** were evaluated for their antiproliferative activity. When the 1,2,3-triazole group was removed, the inhibitory activity against all the cancer cell lines was decreased (IC_50_ > 100 μM), indicating the 1,2,3-triazole scaffold is necessary for inhibitory activity. Especially, compound **9f** showed the most potent inhibitory effect against all cell lines with IC_50_ values ranging from 0.8 μM to 1.7 μM. 

In addition, the substituent on the phenothiazine ring may be important for antiproliferative activity. When the hydrogen atom of compounds **9a**–**9f** was replaced by a trifluoromethyl group of compounds **9g**–**9k,** the antiproliferative activity was decreased. Compound **9g** displayed the weak antiproliferative activity against MCF-7 cells (>100 μM), indicating the significance of the hydrogen atom on the phenothiazine ring for their antiproliferative activity. 

**Figure 5:**
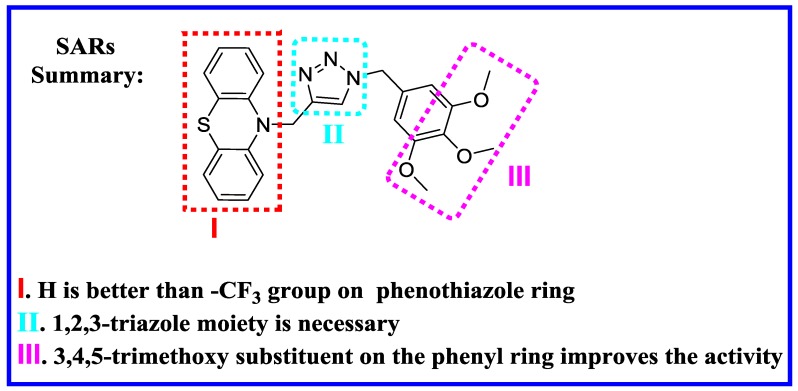
The summary of structure activity relationships.

The substituent on the phenyl ring also displayed a remarkable effect on the antiproliferative activity against the breast cancer cell lines. Compound **9c** with the 4-bromine atom on the phenyl ring showed more potent antiproliferative activity (9.0 μM) than compound **9a** with a 4-F substituent (15.7 μM) against MDA-MB-468 cells, and compound **9f** with a 3,4,5-trimethoxy substituent on the phenyl ring showed more potent antiproliferative activity (0.8 μM) than compound **9d** with the 4-CH_3_ substituent (7.3 μM) against MCF-7 cell line. When 4-CH_3_ substituent (**9d**) as an electron-donating group was replaced by the electron-withdrawing groups like F, Cl, Br (**9a**, **9b**, **9c**), the antiproliferative activity was decreased against all cancer cell lines. The summary of structure activity relationships is shown in Figure 5.

### 2.3. Compound **9f** Induced Morphological Changes of Mcf-7 Cells

**Figure 6:**
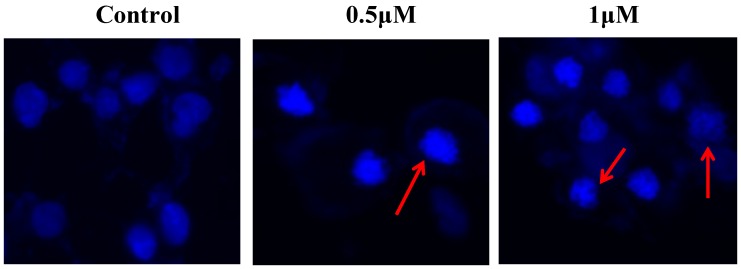
MCF-7 cells treated by **9f** showed typical apoptotic morphologies.

Because of the most potent anticancer activity in vitro against all the selected cancer cell lines among phenothiazine-1,2,3-triazole hybrids, the antiproliferative mechanism of compound **9f** was explored. To explore cytotoxicity of **9f** in MCF-7 cells, cell apoptosis was studied by the reported Hoechst 33258 staining assay [18]. After 24 h incubation with **9f** at 0.5 μM and 1 μM, characteristic apoptotic morphological changes could be served by fluorescence microscope, including cell rounding, chromain shrinkage and formation of apoptotic bodies (Figure 6). Therefore, target compound **9f** could cause morphological changes of MCF-7 cancer cells possibly by inducing apoptosis process.

### 2.4. Compound **9f** Induced Apoptosis of Mcf-7 Cells

**Figure 7:**
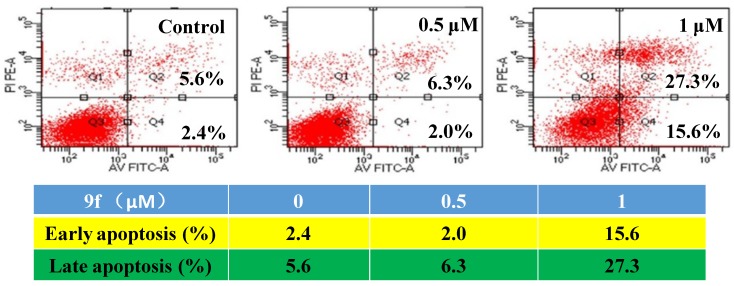
Compound **9f** could induce apoptosis of MCF-7 cells.

Based on the biological experiments above, the apoptosis property of compound **9f** was investigated against MCF-7 cells (Figure 7). After treatment with different concentrations of compound **9f** (0, 0.5 and 1 μM) for 24 h, MCF-7 cells were stained with propidium Iodide and Annexin V Binding Buffer, and then analyzed by the flow cytometry. As illustrated in Figure 7, the percentage of apoptotic cells by treatment of 1 μM compound **9f** was changed obviously from the control group. 

### 2.5. Compound **9f** Related Apoptosis-Related Proteins

The changes of morphological features, such as cell shrinkage, chromatin condensation, nuclear membrane blebbing are the characteristics of apoptotic cells [19]. Based on the above results, we explored the effect of compound **9f** on celluar apoptosis. Bcl-2 family proteins could dictate death decisions or cellular survival by regulating the integrity of the mitochondrial outer membrane [20]. Recent years, Bcl-2 family proteins have emerged as anticancer targets [21]. 

**Figure 8:**
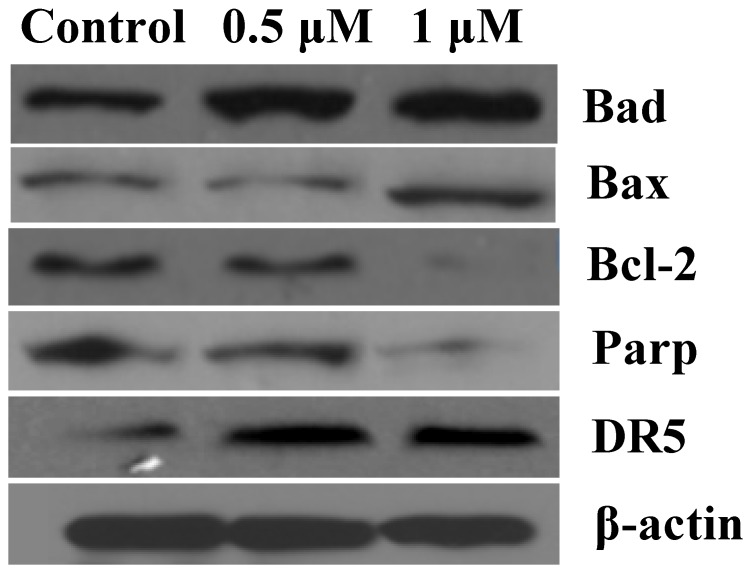
Expression of apoptosis-related proteins in MCF-7 cells after treatment by compound **9f** for 24 h.

Due to the potent cytotoxic activity against MCF-7 cell line, compound **9f** was evaluated the related-apoptosis proteins. In western blot assay, compound **9f** increased the level of Bad, Bax and DR5 and reduced the level of Bcl-2 and Parp which could induce apoptosis of MCF-7 cells (Figure 8). 

## 3. Experimental Section

### 3.1. Chemistry

Reagents and solvents used were purchased from Beijing Research Biotechnology Co., Ltd. (Beijing, China). All NMR spectra were obtained from a Bruker DPX 400 MHz spectrometer (Agilent, Santa Clara, CA, USA) (internal standard was TMS). Mass spectra (MS) were recorded on Esquire 3000 mass spectrometer by electrospray ionization (ESI) (Varian, Palo Alto, CA, USA) for all phenothiazole-1,2,3-triazole hybrids.

#### 3.1.1. Phenothiazine Derivatives **8a**–**8b**

To a stirred solution of phenothiazine or 2-(trifluoromethyl)-10*H*-phenothiazine (3 mmol) in dichloromethane (20 mL), K_2_CO_3_ (5 mmol) and propargyl bromide (5 mmol) were added carefully and the reaction mixture was refluxed for 7 h. After the reaction, the system was concentrated under vacuum, the residue was dissolved in EtOAc (40 mL) and washed by pure water and brine, and dried over anhydrous Na_2_SO_4_ and concentrated under vacuum to afford crude products **8a**–**8b**, which were used in the next reaction without further purification.

#### 3.1.2. General Method to Synthesize Phenothiazine-1,2,3-Triazole Hybrids **9a**–**9k**

Alkyne intermediates **8a**–**8b** (3mmol), azide derivatives **7a**–**7h** (6 mmol), CuSO_4_.5H_2_O (1.2 mmol) and sodium ascorbate (0.6 mmol) were dissolved in THF/H_2_O (20 mL/20 mL). The system was reacted for 8 h at room temperature. After the reaction, the product was filtered to afford the products **9a**–**9k**, which were purified with column chromatography on silica gel (hexane/EtOAc = 9/1).

*10-{[1-(4-Fluorobenzyl)-1H-1,2,3-triazol-4-yl]methyl}-10H-phenothiazine* (**9a**). Yield: 87%. White solid. Mp: 166–167 °C. ^1^H NMR (400 MHz, DMSO-d_6_) δ 8.01 (s, 1H, CHN), 7.29–7.21 (m, 2H, Ar), 7.19–7.06 (m, 6H, Ar), 6.99–6.85 (m, 4H, Ar), 5.54 (s, 2H, PhCH_2_), 5.14 (s, 2H, NCH_2_). ^13^C NMR (100 MHz, DMSO-d_6_) δ 162.96, 160.53, 144.02, 143.77, 132.42, 132.39, 129.87, 129.78, 127.42, 126.74, 123.64, 122.64, 122.62, 115.71, 115.55, 115.34, 51.86, 43.86. HR-MS (ESI): Calcd. C_22_H_18_FN_4_S, [M + H]^+^ *m*/*z*: 389.1236, found: 389.1239.

*10-{[1-(4-Chlorobenzyl)-1H-1,2,3-triazol-4-yl]methyl}-10H-phenothiazine* (**9b**). Yield: 69%. White solid. Mp: 150–151 °C. ^1^H NMR (400 MHz, DMSO-d_6_) δ 8.02 (s, 1H, CHN), 7.57–7.46 (m, 2H, Ar), 7.19–7.04 (m, 6H, Ar), 6.92 (t, *J* = 7.3 Hz, 4H, Ar), 5.54 (s, 2H, PhCH_2_), 5.15 (s, 2H, NCH_2_). ^13^C NMR (100 MHz, DMSO-d_6_) δ 144.01, 143.80, 135.60, 131.53, 129.75, 127.43, 126.75, 123.79, 122.65, 122.63, 121.19, 115.71, 51.91, 43.87. HR-MS (ESI): Calcd. C_22_H_18_ClN_4_S, [M + H]^+^ *m*/*z*: 405.0941, found: 405.0948.

*10-{[1-(4-Bromobenzyl)-1H-1,2,3-triazol-4-yl]methyl}-10H-phenothiazine* (**9c**). Yield: 77%. White solid. Mp: 154–155 °C. ^1^H NMR (400 MHz, DMSO-d_6_) δ 8.02 (s, 1H, CHN), 7.49–7.28 (m, 2H, Ar), 7.19 (d, *J* = 8.5 Hz, 2H, Ar), 7.16–7.05 (m, 4H, Ar), 6.93 (t, *J* = 7.2 Hz, 4H, Ar), 5.56 (s, 2H, PhCH_2_), 5.15 (s, 2H, NCH_2_). ^13^C NMR (100 MHz, DMSO-d_6_) δ 144.01, 143.81, 135.18, 132.66, 129.44, 128.61, 127.43, 126.75, 123.78, 122.65, 122.63, 115.71, 51.85, 43.86. HR-MS (ESI): Calcd. C_22_H_18_BrN_4_S, [M + H]^+^ *m*/*z*: 449.0436, found: 449.0440.

*10-{[1-(4-Methylbenzyl)-1H-1,2,3-triazol-4-yl]methyl}-10H-phenothiazine* (**9d**). Yield: 90%. White solid. Mp: 151–152 °C. ^1^H NMR (400 MHz, DMSO-d_6_) δ 7.98 (s, 1H, CHN), 7.23–7.00 (m, 8H, Ar), 7.00–6.79 (m, 4H, Ar), 5.49 (s, 2H, PhCH_2_), 5.13 (s, 2H, NCH_2_), 2.26 (s, 3H, CH_3_). ^13^C NMR (100 MHz, DMSO-d_6_) δ 144.03, 143.65, 137.25, 133.15, 129.14, 127.53, 127.41, 126.73, 123.57, 122.61, 115.70, 52.45, 43.86, 20.63. HR-MS (ESI): Calcd. C_23_H_21_N_4_S, [M + H]^+^ *m*/*z*: 385.1487, found: 385.1489.

*10-{[1-Benzyl-1H-1,2,3-triazol-4-yl]methyl}-10H-phenothiazine* (**9e**). Yield: 82%. White solid. Mp: 150–152 °C. ^1^H NMR (400 MHz, DMSO-d_6_) δ 8.01 (s, 1H, CHN), 7.37–7.23 (m, 3H, Ar), 7.20–7.05 (m, 6H, Ar), 6.98–6.87 (m, 4H, Ar), 5.55 (s, 2H, PhCH_2_), 5.15 (s, 2H, NCH_2_). ^13^C NMR (100 MHz, DMSO-d_6_) δ 144.03, 143.73, 136.18, 128.61, 127.92, 127.45, 127.41, 126.74, 123.75, 122.65, 122.62, 115.72, 52.63, 43.87. HR-MS (ESI): Calcd. C_22_H_19_N_4_S, [M + H]^+^ *m*/*z*: 371.1330, found: 371.1339.

*10-{[1-(3,4,5-Trimethoxybenzyl)-1H-1,2,3-triazol-4-yl]methyl}-10H-phenothiazine* (**9f**). Yield: 88%. White solid. Mp: 146–147 °C. ^1^H NMR (400 MHz, DMSO-d_6_) δ 8.05 (s, 1H, CHN), 7.19–7.01 (m, 4H, Ar), 7.01–6.83 (m, 4H, Ar), 6.55 (s, 2H, Ar), 5.46 (s, 2H, PhCH_2_), 5.14 (s, 2H, NCH_2_), 3.67 (s, 6H, OCH_3_), 3.62 (s, 3H, OCH_3_). ^13^C NMR (100 MHz, DMSO-d_6_) δ 152.92, 144.03, 143.66, 137.12, 131.56, 127.39, 126.75, 123.58, 122.62, 122.52, 115.64, 105.12, 59.94, 55.80, 52.87, 43.80. HR-MS (ESI): Calcd. C_25_H_25_N_4_O_3_S, [M+Na]^+^ *m*/*z*: 483.1467, found: 483.1468.

*10-{[1-(4-Bromophenyl)-1H-1,2,3-triazol-4-yl]methyl}-2-{trifluoromethyl}-10H-phenothiazine* (**9g**). Yield: 93%. White solid. Mp: 161–162 °C. ^1^H NMR (400 MHz, DMSO-d_6_) δ 8.84 (s, 1H, CHN), 7.86 (d, *J* = 8.9 Hz, 2H, Ar), 7.78 (d, *J* = 8.9 Hz, 2H, Ar), 7.35 (d, *J* = 8.0 Hz, 1H, Ar), 7.26 (d, *J* = 7.5 Hz, 2H, Ar), 7.21–7.12 (m, 2H, Ar), 7.04 (d, *J* = 8.0 Hz, 1H, Ar), 6.98 (t, *J* = 7.3 Hz, 1H, Ar), 5.30 (s, 2H, NCH_2_). ^13^C NMR (100 MHz, DMSO-d_6_) δ 144.58, 144.35, 143.29, 135.63, 132.76, 128.40, 128.22, 127.97, 127.32, 126.92, 125.41, 123.36, 122.70, 121.73 (d, *J* = 14.8 Hz), 121.29, 119.21, 116.16, 112.03, 43.69. HR-MS (ESI): Calcd. C_22_H_15_BrF_3_N_4_S, [M + H]^+^ *m*/*z*: 503.0153, found: 503.0158. 

*10-{[1-(4-Chlorophenyl)-1H-1,2,3-triazol-4-yl]methyl}-2-{trifluoromethyl}-10H-phenothiazine* (**9h**). Yield: 90%. White solid. Mp: 153–155 °C. ^1^H NMR (400 MHz, DMSO-d_6_) δ 8.84 (s, 1H, CHN), 7.99–7.86 (m, 2H, Ar), 7.73–7.56 (m, 2H, Ar), 7.35 (d, *J* = 7.7 Hz, 1H, Ar), 7.26 (d, *J* = 8.4 Hz, 2H, Ar), 7.21–7.12 (m, 2H, Ar), 7.04 (d, *J* = 7.9 Hz, 1H, Ar), 6.99 (t, *J* = 7.4 Hz, 1H, Ar), 5.31 (s, 2H, NCH_2_). ^13^C NMR (100 MHz, DMSO-d_6_) δ 144.58, 144.33, 143.30, 135.23, 132.93, 129.83, 128.41, 128.22, 127.97, 127.91, 127.31, 126.92, 123.36, 121.83, 121.66, 121.56, 119.20, 116.16, 112.03, 43.58. HR-MS (ESI): Calcd. C_22_H_15_ClF_3_N_4_S, [M + H]^+^ *m*/*z*: 459.0658, found: 459.0662. 

*10-{[1-(4-Fluorobenzyl)-1H-1,2,3-triazol-4-yl]methyl}-2-{trifluoromethyl}-10H-phenothiazine* (**9i**). Yield: 81%. White solid. Mp: 134–136 °C. ^1^H NMR (400 MHz, DMSO-d_6_) δ 8.07 (s, 1H, CHN), 7.33 (d, *J* = 7.9 Hz, 1H, Ar), 7.28–7.19 (m, 3H, Ar), 7.19–7.07 (m, 5H, Ar), 7.06–6.90 (m, 2H, Ar), 5.56 (s, 2H, PhCH_2_), 5.21 (s, 2H, NCH_2_). ^13^C NMR (100 MHz, DMSO-d_6_) δ 144.44, 143.37, 143.27, 132.41, 132.38, 129.75, 129.66, 127.92, 127.30, 126.90, 125.35, 123.68, 123.31, 121.53, 119.16, 116.11, 115.49, 115.28, 111.93, 51.89, 43.88. HR-MS (ESI): Calcd. C_23_H_17_F_4_N_4_S, [M + H]^+^ *m*/*z*: 457.1110, found: 457.1118.

*10-{[1-Benzyl-1H-1,2,3-triazol-4-yl]methyl}-2-{trifluoromethyl}-10H-phenothiazine* (**9j**). Yield: 79%. White solid. Mp: 138–139 °C. ^1^H NMR (400 MHz, DMSO-d_6_) δ 8.07 (s, 1H, CHN), 7.39–7.27 (m, 4H, Ar), 7.24 (d, *J* = 8.1 Hz, 1H, Ar), 7.20–7.07 (m, 5H, Ar), 7.03–6.93 (m, 2H, Ar), 5.58 (s, 2H, PhCH_2_), 5.22 (s, 2H, NCH_2_). ^13^C NMR (100 MHz, DMSO-d_6_) δ 144.45, 143.38, 143.21, 136.19, 128.56, 128.24, 128.18, 127.90, 127.30, 127.24, 126.89, 125.37, 123.82, 123.31, 122.66, 121.51, 119.08, 116.12, 111.97, 52.62, 43.92. HR-MS (ESI): Calcd. C_23_H_18_F_3_N_4_S, [M + H]^+^ *m*/*z*: 439.1204, found: 439.1208. 

*10-{[1-(4-Chlorobenzyl)-1H-1,2,3-triazol-4-yl]methyl}-2-{trifluoromethyl}-10H-phenothiazine* (**9k**). Yield: 86%. White solid. Mp: 140–142 °C. ^1^H NMR (400 MHz, DMSO-d_6_) δ 8.08 (s, 1H, CHN), 7.43–7.28 (m, 3H, Ar), 7.24 (d, *J* = 8.0 Hz, 1H, Ar), 7.17 (ddd, *J* = 5.7, 3.8, 2.4 Hz, 4H, Ar), 7.12 (s, 1H, Ar), 7.05–6.85 (m, 2H, Ar), 5.58 (s, 2H, PhCH_2_), 5.22 (s, 2H, NCH_2_). ^13^C NMR (100 MHz, DMSO-d_6_) δ 144.44, 143.31, 135.17, 132.66, 129.31, 128.54, 128.27, 128.17, 127.92, 127.85, 127.31, 126.90, 123.82, 123.31, 121.55, 119.13, 119.09. 116.12, 111.89, 51.88, 43.89. HR-MS (ESI): Calcd. C_23_H_17_ClF_3_N_4_S, [M + H]^+^ *m*/*z*: 473.0815, found: 473.0819. This compound was also reported in the previous paper [22].

### 3.2. Biology

#### 3.2.1. Antiproliferative Activity 

The all breast cancer cell lines were maintained in the mixture of 89% RPMI1640 medium, 10% fetal bovine serum and 1% penicillinestreptomycin. All cell lines were purchased from the China Center for Type Culture Collection (Shanghai, China). The cancer cells were seeded into 96-well plates (5000 cells per well). After 24 h incubation, the culture medium was removed and replaced with medium containing the candidate compounds. The cells were incubated for another 72 h. Then, 30 µL (3-(4,5-dimethylthiazol-2-yl)-2,5-diphenyltetrazo lium bromide) solution (5 mg/mL) was added and incubated for 2 h. Dimethyl sulfoxide (40 µL) was added to each well to dissolve the dark blue formazan; the absorbance was measured using a microplate reader at awavelength of 570 nm. 

#### 3.2.2. Apoptosis Analysis with Hoechst 33258 Staining

Near-confluent MCF-7 cells were seeded into a 6-well plate (5.0 × 10^6^ cells/well). After incubation for 24 h with the test compound, Hoechst 33258 was added to the culture medium to give final concentrations of 5 mg/mL, respectively. The cells were incubated with the staining mixture for 30 min at 37 °C and then the cells were observed under a fluorescence microscope (Leica Microsystems AG, Wetzlar, Germany). Apoptotic cells were examined and identified according to the condensation and fragmentation of their nuclei by fluorescence microscopy. Hoechst 33258 permeates all the cells and makes the nuclei appear blue. Apoptosis was revealed by nuclear changes such as chromatin condensation and nuclear fragmentation. 

#### 3.2.3. Apoptosis Analysis with Western Blot

MCF-7 cells were cultured with different concentrations of compound **9f** for 24 h, both adherent and floating cells were collected, and then Western blot analysis was performed. Briefly, Cells were lysed in cell lysis buffer [1% NP-40, 0.1% sodium dodecyl sulfate (SDS), 150 mM NaCl, 25 mM TriseHCl, 1% deoxycholic acid sodium salt, 1% PMSF] for 30 min on ice. Total protein contents were measured by protein assay reagent. The membrane was indicated primary antibodies at 4 °C. After that, the membrane was incubated with secondary antibodies, goat anti-rabbit and goat anti-mouse IgG conjugated. 

## 4. Conclusions

We designed and synthesized a series of novel phenothiazine-1,2,3-triazole hybrids and evaluated their anticancer activity in vitro against three selected cancer cell lines (MDA-MB-468, MDA-MB-231 and MCF-7). Among these hybrids, compound **9f** showed the most excellent anticancer activity in vitro with an IC_50_ value of 0.8 μM against MCF-7 cancer cells. Biological mechanism displayed that **9f** induced morphological change and apoptosis against MCF-7 cells by increasing the level of Bad, Bax and DR5 and decreasing the level of Bcl-2 and Parp. 

## Figures and Tables

**Table 1:** Antiproliferative results of phenothiazine-1,2,3-triazole hybrids **9a**–**9k**.

Compound	IC_50_ (μM) ^a^		
	MDA-MB-231	MDA-MB-468	MCF-7
**8a**	>100	>100	>100
**8b**	>100	>100	>100
**9a**	9.0 ± 0.6	15.7 ± 0.3	8.6 ± 1.1
**9** **b**	11.4 ± 0.2	5.5 ± 1.7	8.8 ± 2.0
**9** **c**	14.2 ± 1.3	9.0 ± 0.9	11.1 ± 0.5
**9** **d**	2.0 ± 0.2	1.6 ± 0.3	7.3 ± 1.1
**9** **e**	10.5 ± 1.0	8.4 ± 0.8	7.2 ± 0.2
**9f**	1.7 ± 0.1	1.2 ± 0.2	0.8 ± 0.1
**9** **g**	56.3 ± 0.8	46.4 ± 2.8	>100
**9** **h**	39.1 ± 0.6	43.3 ± 0.7	29.6 ± 1.3
**9** **i**	11.3 ± 2.6	11.2 ± 0.8	14.2 ± 1.7
**9** **j**	17.4 ± 1.4	17.6 ± 2.4	15.3 ± 0.4
**9** **k**	17.3 ± 0.9	14.3 ± 0.8	16.4 ± 1.1
**5-Fu**	10.8 ± 0.3	7.5 ± 0.2	12.7 ± 0.6

^a^ Inhibitory activity was assayed by exposure for 72 h to substances and expressed as concentration required to inhibit tumor cell proliferation by 50% (IC_50_). Data are presented as the means ± SDs of three independent experiments.

## Supplementary Materials

The spectra of compounds **9a**–**9k** are available online.
