# Supplementary material for: Antiproliferative Phenothiazine Hybrids as Novel Apoptosis Inducers against MCF-7 Breast Cancer
Source: Molecules. 2018 May 28;23(6):1288. doi: 10.3390/molecules23061288 (PMC6100384; doi:10.3390/molecules23061288)

Article

Antiproliferative Phenothiazine Hybrids as Novel Apoptosis Inducers against MCF-7 Breast Cancer

Jun-Xia Zhang [1,2], Jiao-Mei Guo [2], Ting-Ting Zhang [2], Hong-Jun Lin [2], Nai-Song Qi [2], Zhen-Guo Li [2], Ji-Chun Zhou [2], Zhen-Zhong Zhang [1,3,*]

1. School of Pharmaceutical science, Zhengzhou University, Zhengzhou 450001, China; zjx312@163.com
2. Department of Pharmacology, Henan Provincial Institute of Food and Drug Control, Zhengzhou 450008, China; ssjsel745288@163.com (J.-M.G.); 18637123006@163.com (T.-T.Z.); 13393723919@163.com (H.-J.L.); 18538293031@163.com (N.-S.Q.); 13298181239@163.com (Z.-G.L.); 18838969948@163.com (J.-C.Z.)
3. Key Laboratory of Targeting Therapy and Diagnosis for Critical Diseases, Henan Province, Zhengzhou 450001, China

* Correspondence: zhangzhenzhong@zzu.edu.cn; Tel.: +0371-63388226

10-((1-(4-Fluorobenzyl)-1H-1,2,3-triazol-4-yl)methyl)-10H-phenothiazine (9a)

Yield: 87%. White solid. Mp: 166~167°C. ^{1}H NMR (400 MHz, DMSO) δ 8.01 (s, 1H), 7.29 – 7.21 (m, 2H), 7.19 – 7.06 (m, 6H), 6.99 – 6.85 (m, 4H), 5.54 (s, 2H), 5.14 (s, 2H). ^{13}C NMR (100 MHz, DMSO) δ 162.96, 160.53, 144.02, 143.77, 132.42, 132.39, 129.87, 129.78, 127.42, 126.74, 123.64, 122.64, 122.62, 115.71, 115.55, 115.34, 51.86, 43.86. HR-MS (ESI): Calcd. $C_{22}H_{18}FN_4S$, $[M+H]^+$m/z: 389.1236, found: 389.1239.

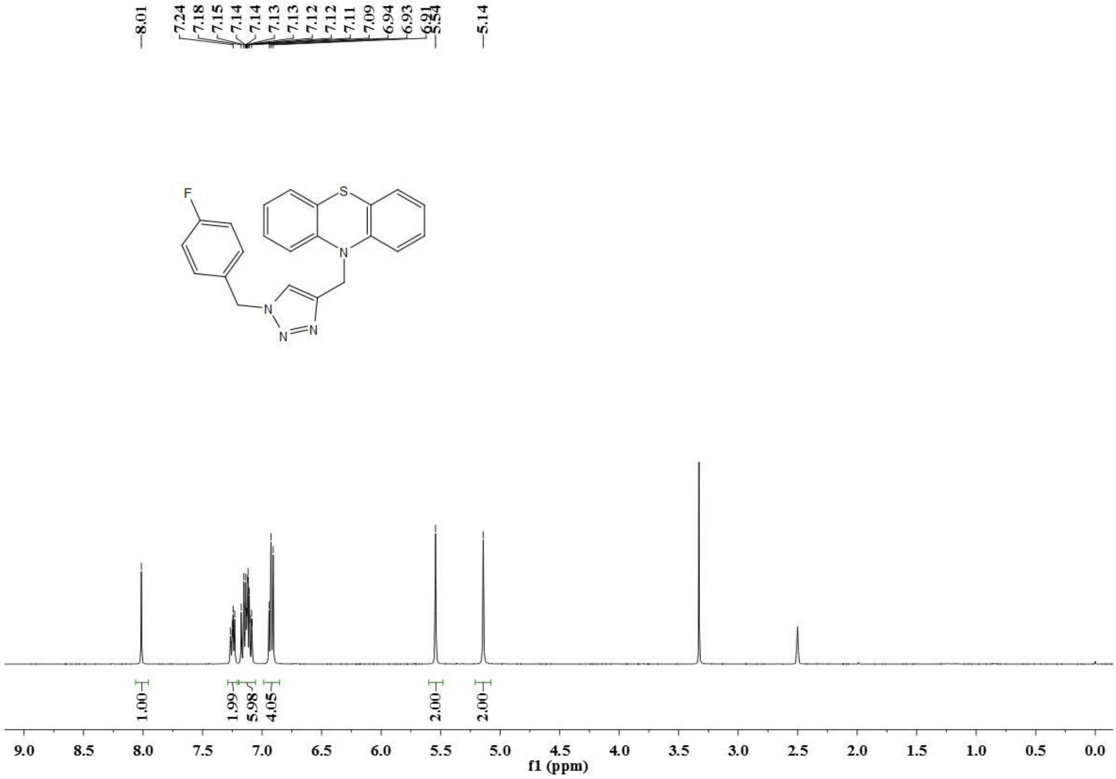

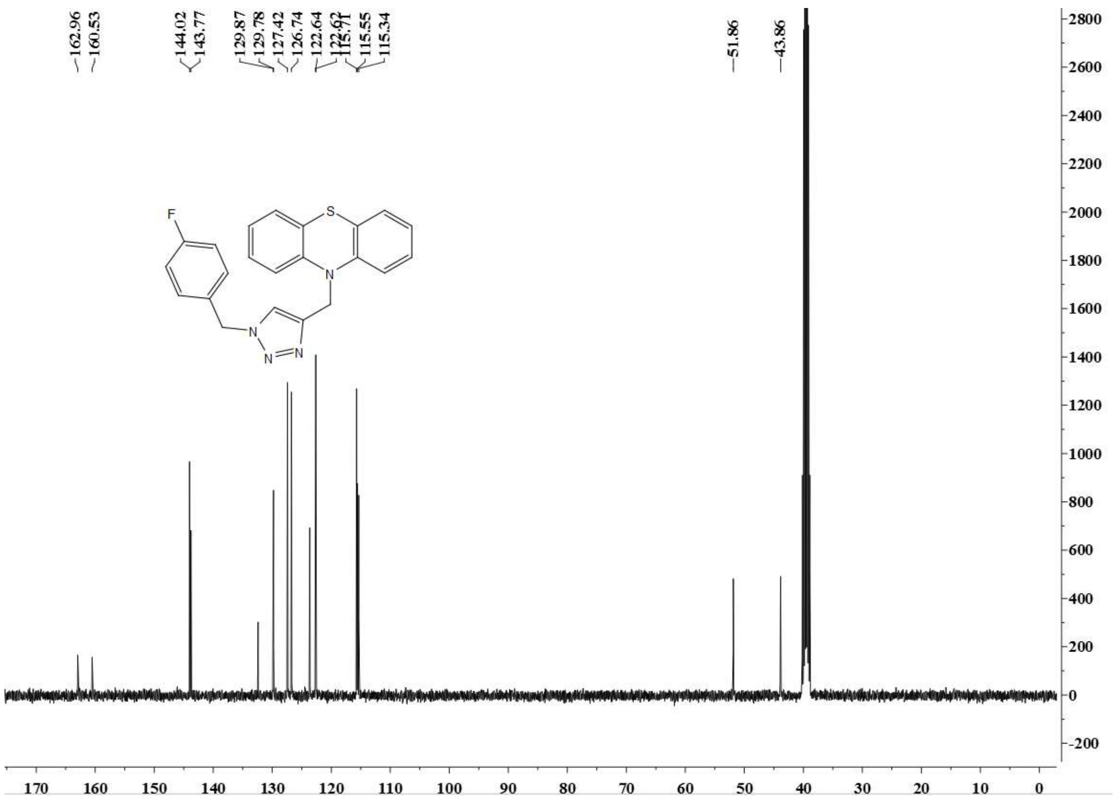

10-((1-(4-Chlorobenzyl)-1H-1,2,3-triazol-4-yl)methyl)-10H-phenothiazine (9b)

Yield: 69%. White solid. Mp: 150~151°C. ^{1}H NMR (400 MHz, DMSO) δ 8.02 (s, 1H), 7.57 – 7.46 (m, 2H), 7.19 – 7.04 (m, 6H), 6.92 (t, *J* = 7.3 Hz, 4H), 5.54 (s, 2H), 5.15 (s, 2H). ^{13}C NMR (100 MHz, DMSO) δ 144.01, 143.80, 135.60, 131.53, 129.75, 127.43, 126.75, 123.79, 122.65, 122.63, 121.19, 115.71, 51.91, 43.87. HR-MS (ESI): Calcd. $C_{22}H_{18}ClN_4S$, $[M+H]^+$m/z: 405.0941, found: 405.0948.

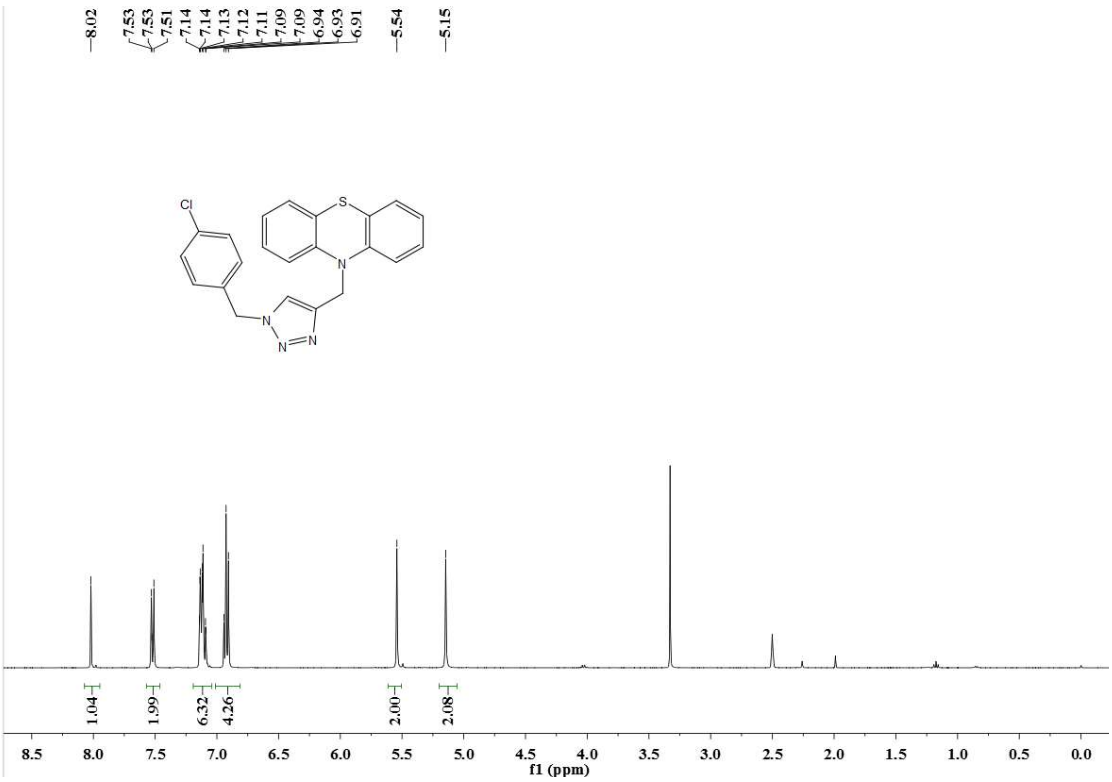

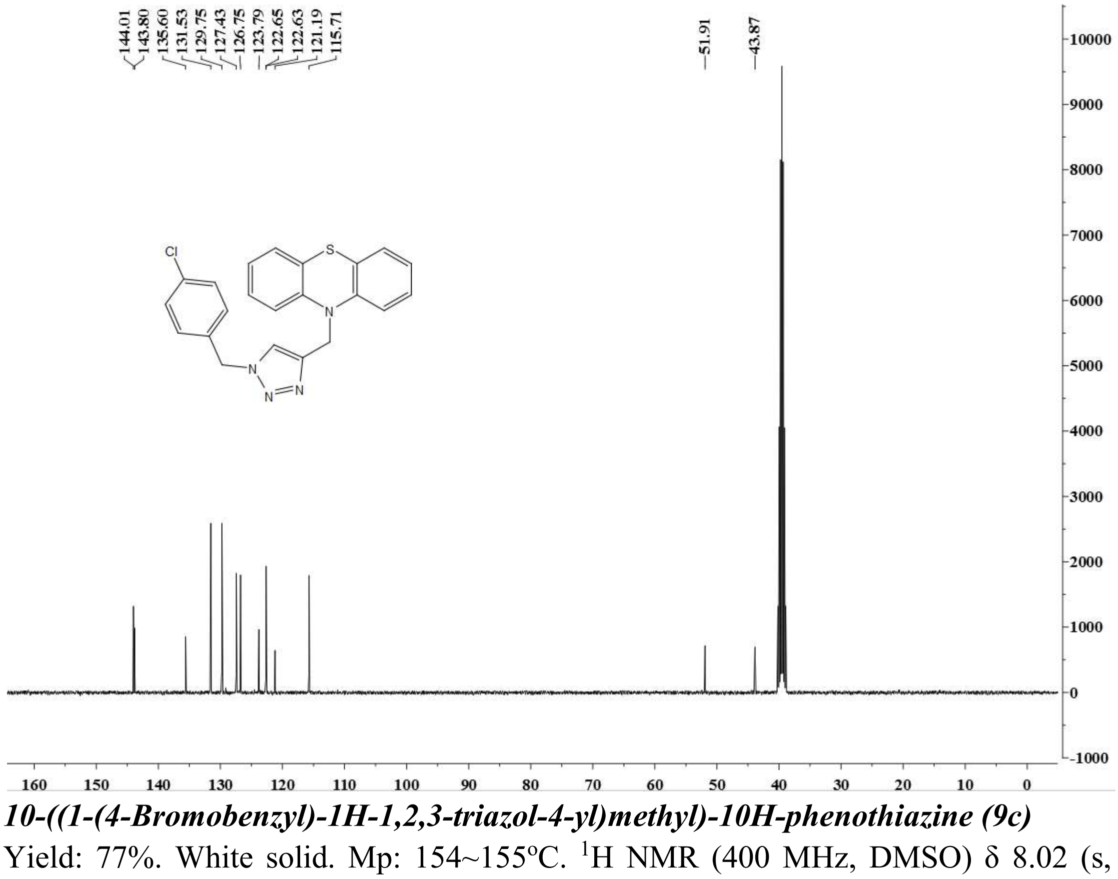

10-((1-(4-Bromobenzyl)-1H-1,2,3-triazol-4-yl)methyl)-10H-phenothiazine (9c)

Yield: 77%. White solid. Mp: 154~155ºC. ^{1}H NMR (400 MHz, DMSO) δ 8.02 (s, 1H), 7.49 – 7.28 (m, 2H), 7.19 (d, *J* = 8.5 Hz, 2H), 7.16 – 7.05 (m, 4H), 6.93 (t, *J* = 7.2 Hz, 4H), 5.56 (s, 2H), 5.15 (s, 2H). ^{13}C NMR (100 MHz, DMSO) δ 144.01, 143.81, 135.18, 132.66, 129.44, 128.61, 127.43, 126.75, 123.78, 122.65, 122.63, 115.71, 51.85, 43.86. HR-MS (ESI): Calcd. $C_{22}H_{18}BrN_4S$, $[M+H]^+$m/z: 449.0436,

found: 449.0440.

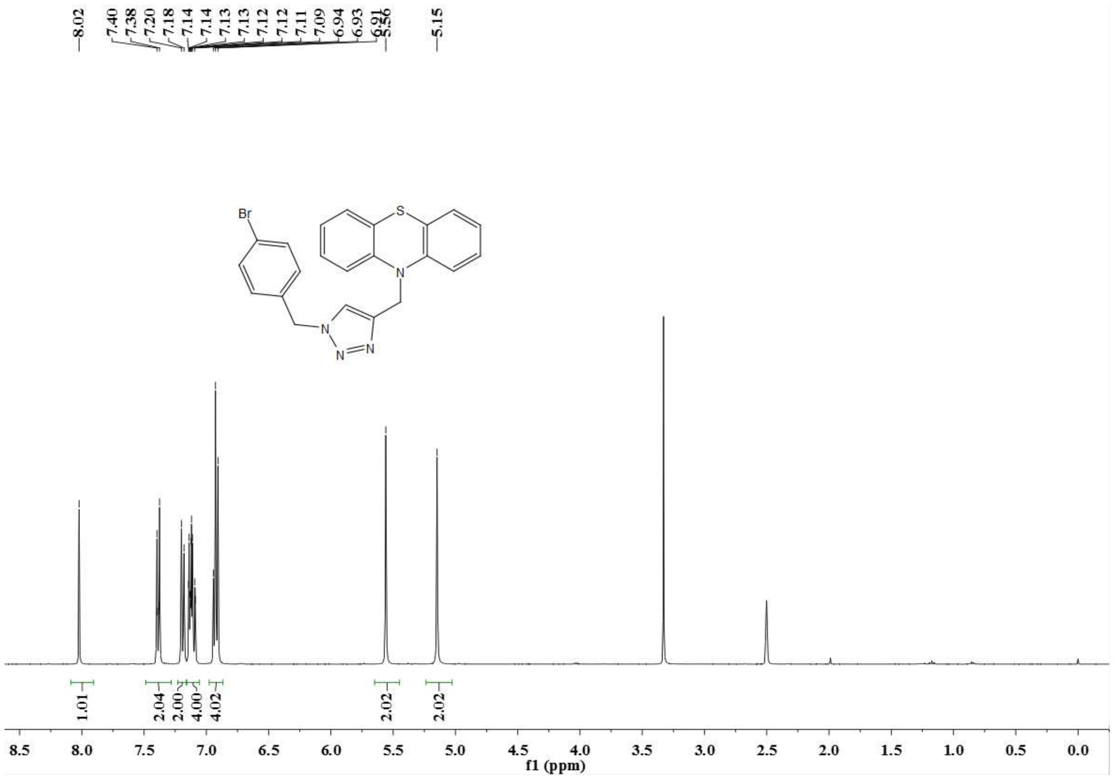

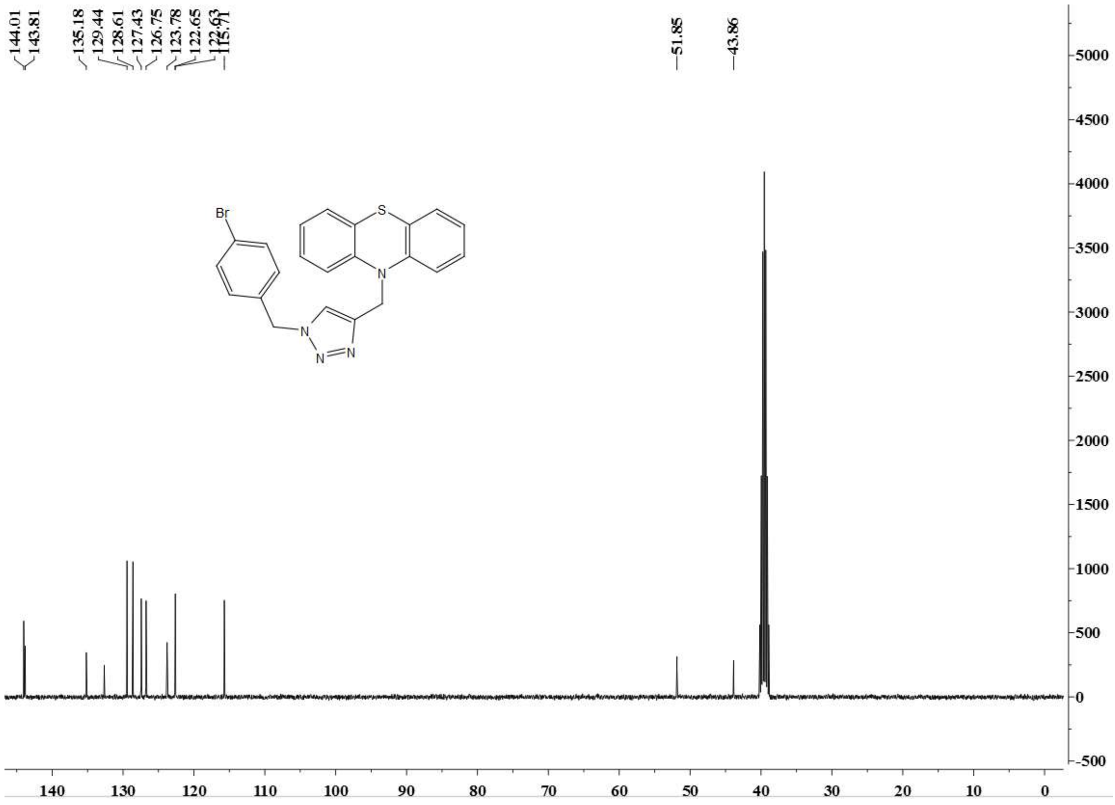

10-((1-(4-Methylbenzyl)-1H-1,2,3-triazol-4-yl)methyl)-10H-phenothiazine (9d)

Yield: 90%. White solid. Mp: 151~152ºC. ^{1}H NMR (400 MHz, DMSO) δ 7.98 (s, 1H), 7.23 – 7.00 (m, 8H), 7.00 – 6.79 (m, 4H), 5.49 (s, 2H), 5.13 (s, 2H), 2.26 (s, 3H). ^{13}C NMR (100 MHz, DMSO) δ 144.03, 143.65, 137.25, 133.15, 129.14, 127.53,

127.41, 126.73, 123.57, 122.61, 115.70, 52.45, 43.86, 20.63. HR-MS (ESI): Calcd. $C_{23}H_{21}N_4S$, $[M+H]^+$m/z: 385.1487, found: 385.1489.

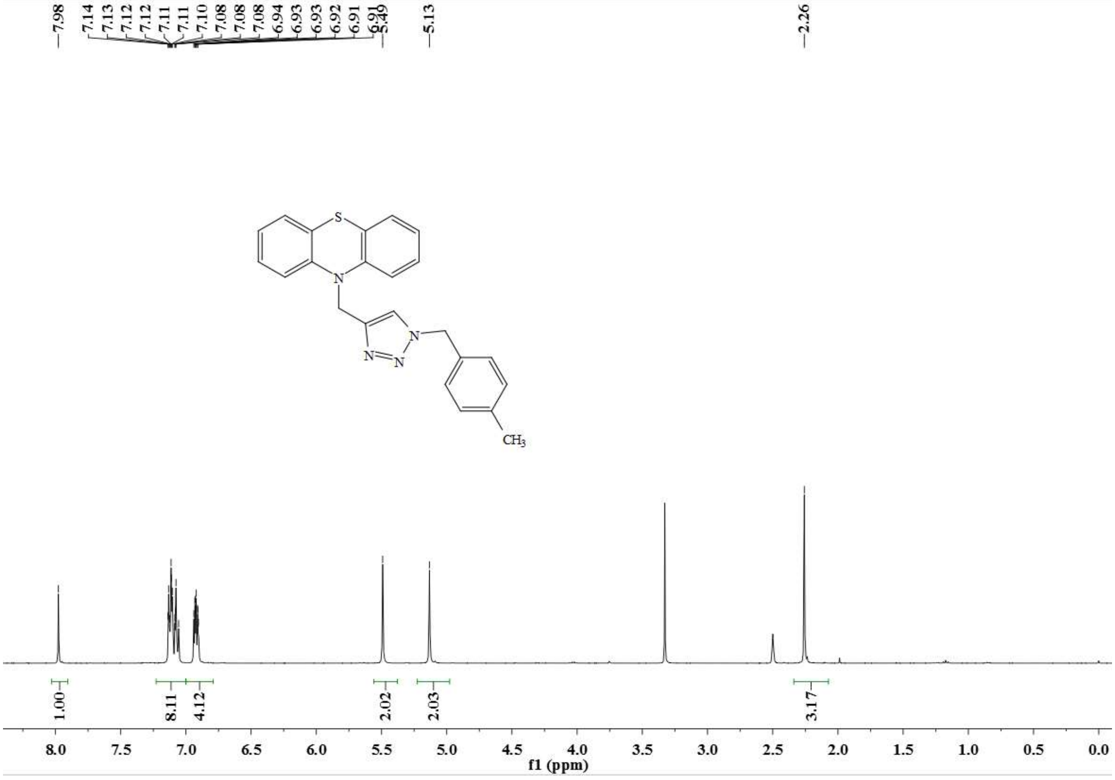

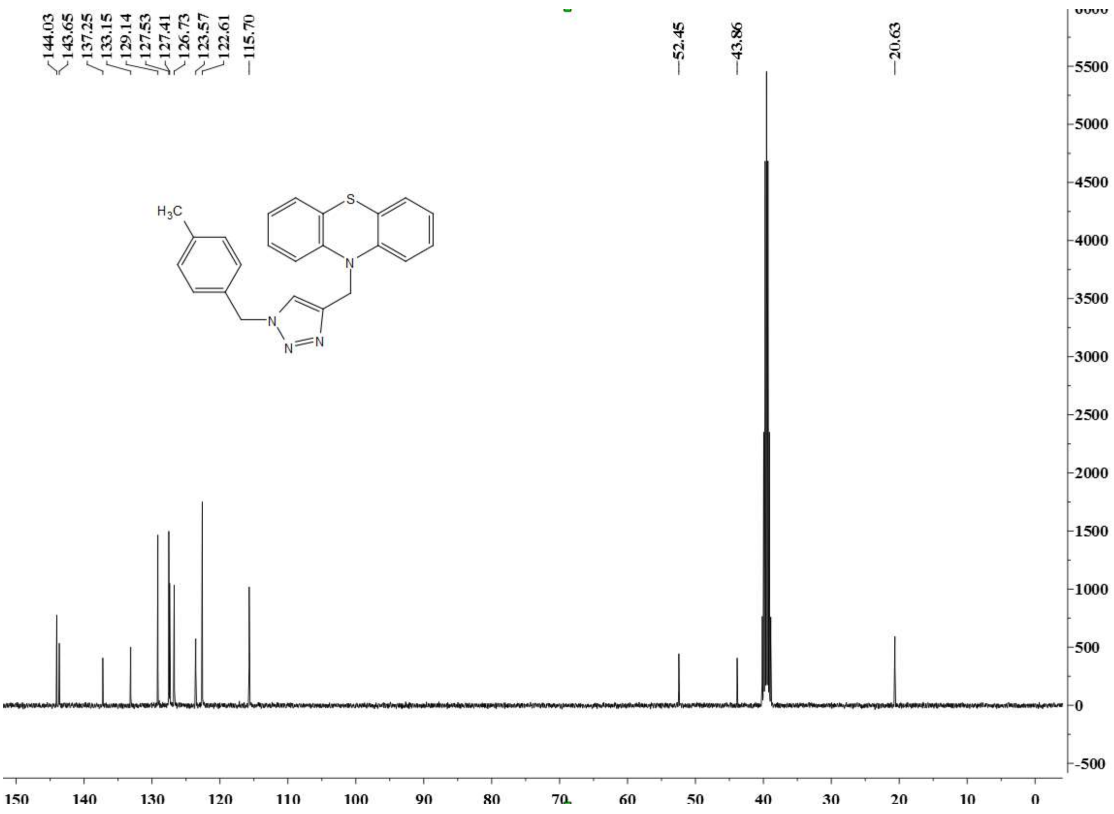

10-((1-Benzyl-1H-1,2,3-triazol-4-yl)methyl)-10H-phenothiazine (9e)

Yield: 82%. White solid. Mp: 150~152ºC. ^{1}H NMR (400 MHz, DMSO) δ 8.01 (s, 1H), 7.37 – 7.23 (m, 3H), 7.20 – 7.05 (m, 6H), 6.98 – 6.87 (m, 4H), 5.55 (s, 2H), 5.15

(s, 2H). ^{13}C NMR (100 MHz, DMSO) δ 144.03, 143.73, 136.18, 128.61, 127.92, 127.45, 127.41, 126.74, 123.75, 122.65, 122.62, 115.72, 52.63, 43.87. HR-MS (ESI): Calcd. $C_{22}H_{19}N_4S$, $[M+H]^+$m/z: 371.1330, found: 371.1339.

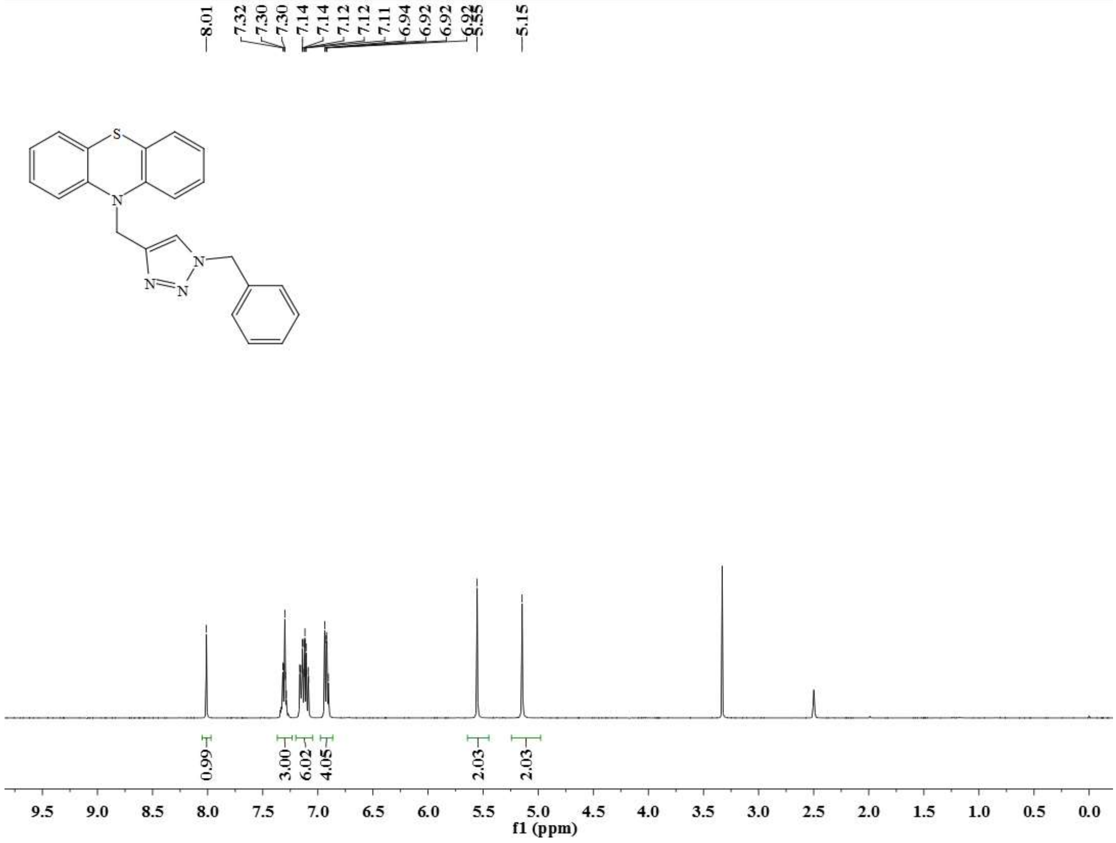

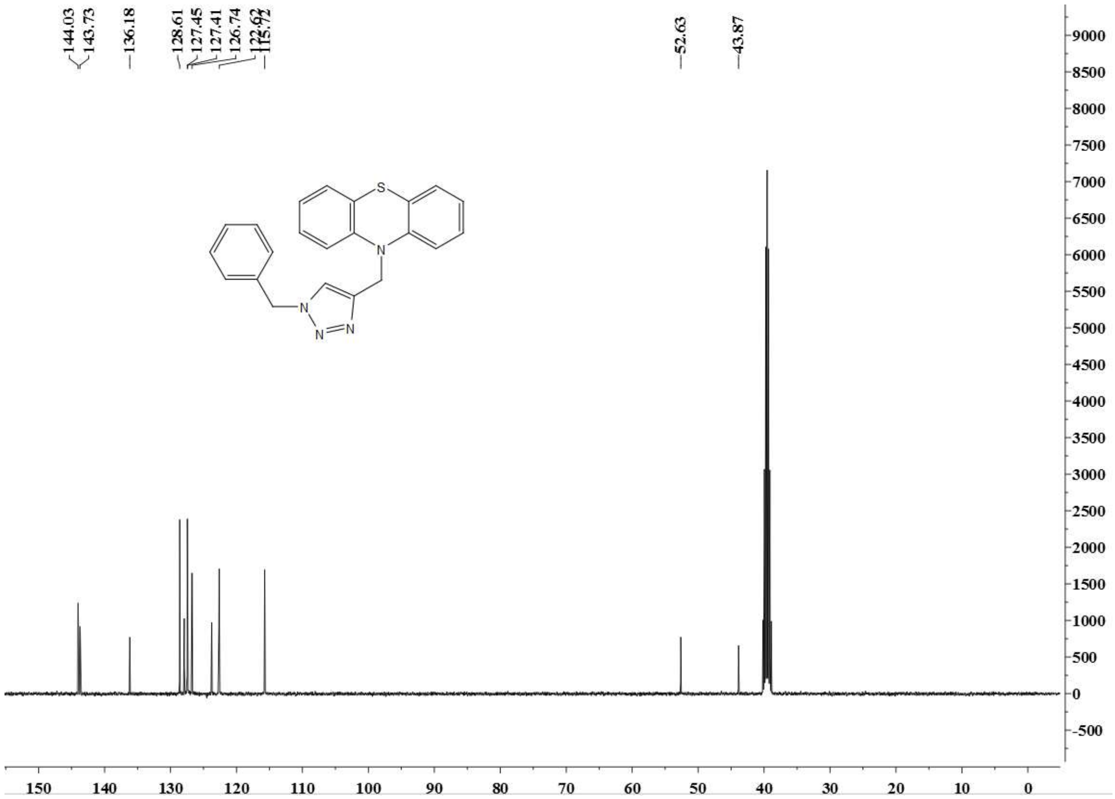

10-((1-(3,4,5-Trimethoxybenzyl)-1H-1,2,3-triazol-4-yl)methyl)-10H-phenothiazine

(9f)

Yield: 88%. White solid. Mp: 146~147°C. ^{1}H NMR (400 MHz, DMSO) δ 8.05 (s, 1H), 7.19 – 7.01 (m, 4H), 7.01 – 6.83 (m, 4H), 6.55 (s, 2H), 5.46 (s, 2H), 5.14 (s, 2H), 3.67 (s, 6H), 3.62 (s, 3H). ^{13}C NMR (100 MHz, DMSO) δ 152.92, 144.03, 143.66, 137.12, 131.56, 127.39, 126.75, 123.58, 122.62, 122.52, 115.64, 105.12, 59.94, 55.80, 52.87, 43.80. HR-MS (ESI): Calcd. $C_{25}H_{25}N_4O_3S$, $[M+H]^+$m/z: 461.1647, found: 461.1649.

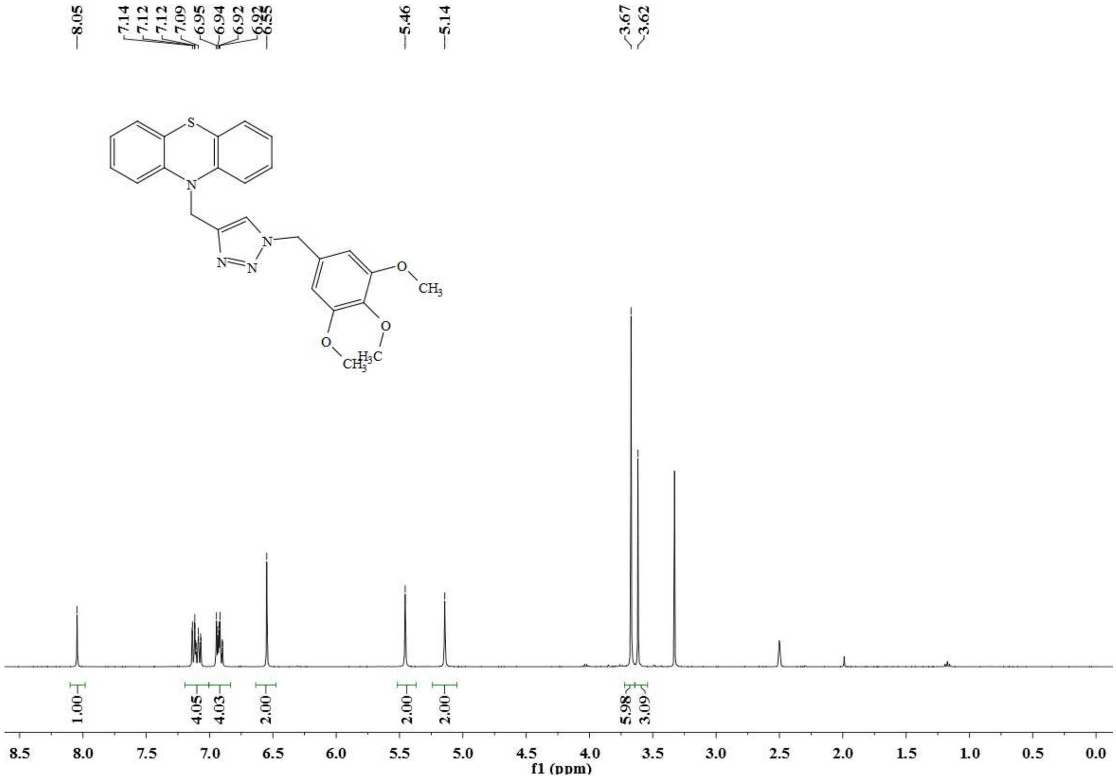

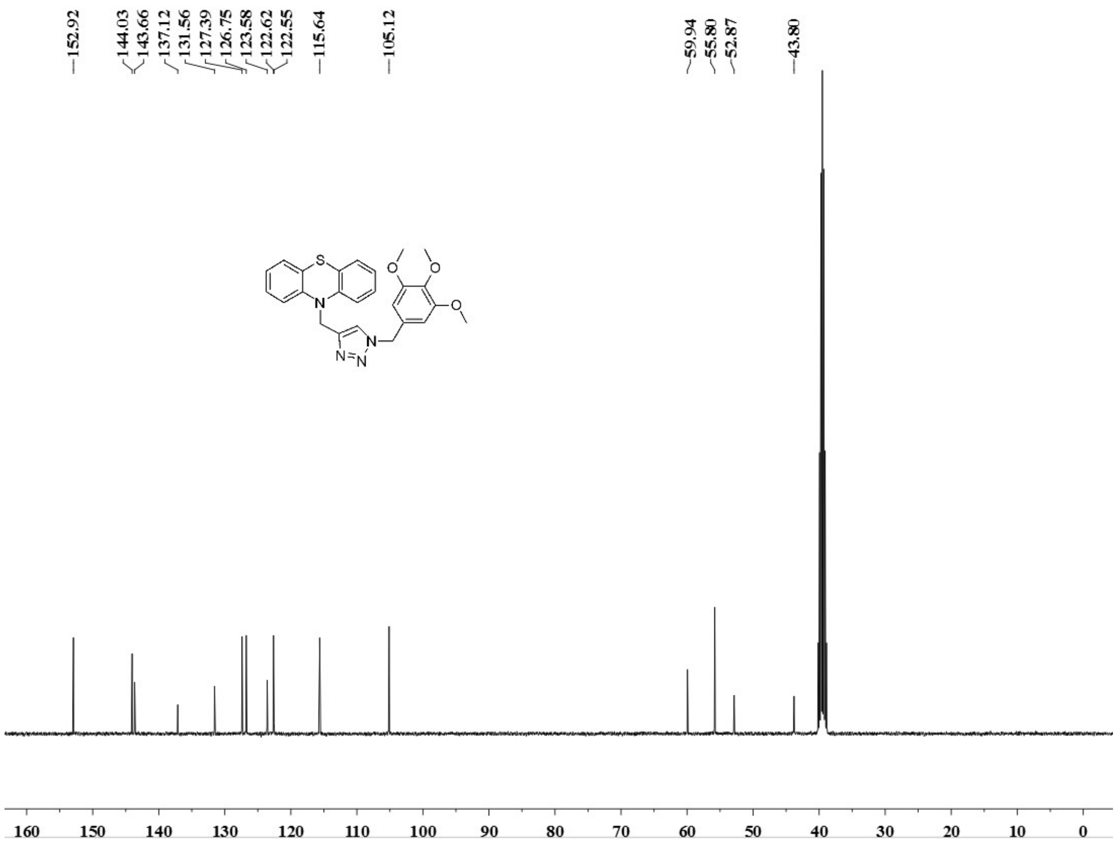

10-((1-(4-Bromophenyl)-1H-1,2,3-triazol-4-yl)methyl)-2-(trifluoromethyl)-10H-phenothiazine (9g)

Yield: 93%. White solid. Mp: 161~162°C. ^{1}H NMR (400 MHz, DMSO) δ 8.84 (s, 1H), 7.86 (d, J = 8.9 Hz, 2H), 7.78 (d, J = 8.9 Hz, 2H), 7.35 (d, J = 8.0 Hz, 1H), 7.26 (d, J = 7.5 Hz, 2H), 7.21 – 7.12 (m, 2H), 7.04 (d, J = 8.0 Hz, 1H), 6.98 (t, J = 7.3 Hz, 1H), 5.30 (s, 2H). ^{13}C NMR (100 MHz, DMSO) δ 144.58, 144.35, 143.29, 135.63, 132.76, 128.40, 128.22, 127.97, 127.32, 126.92, 125.41, 123.36, 122.70, 121.73 (d, J = 14.8 Hz), 121.29, 119.21, 116.16, 112.03, 43.69. HR-MS (ESI): Calcd. $C_{22}H_{15}BrF_3N_4S$, $[M+H]^+$m/z: 503.0153, found: 503.0158.

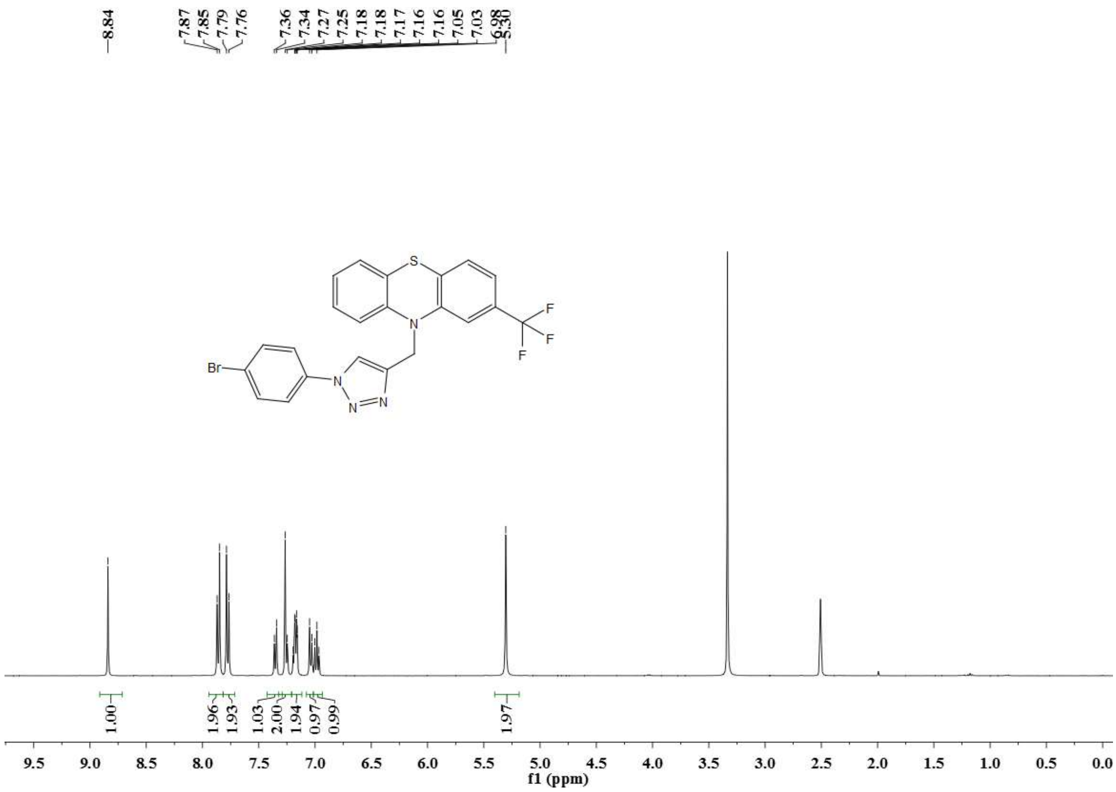

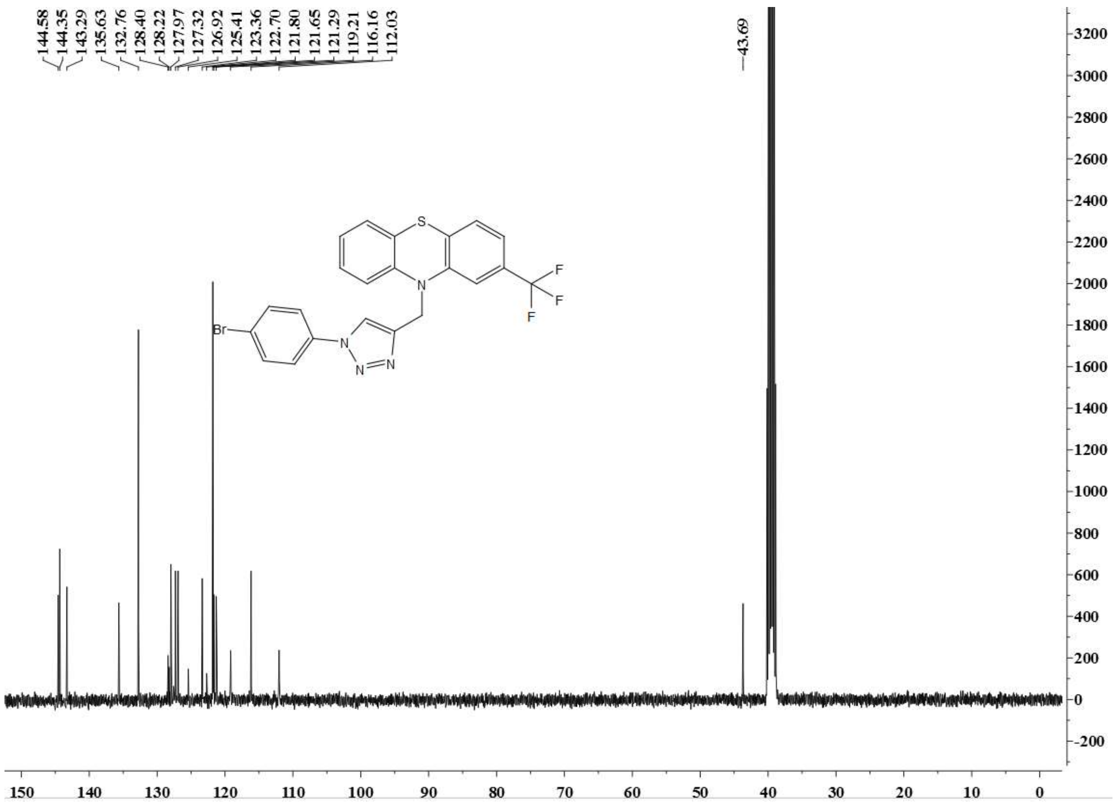

10-((1-(4-Chlorophenyl)-1H-1,2,3-triazol-4-yl)methyl)-2-(trifluoromethyl)-10H-phenothiazine (9h)

Yield: 90%. White solid. Mp: 153~155ºC. ^{1}H NMR (400 MHz, DMSO) δ 8.84 (s,

1H), 7.99 – 7.86 (m, 2H), 7.73 – 7.56 (m, 2H), 7.35 (d, J = 7.7 Hz, 1H), 7.26 (d, J = 8.4 Hz, 2H), 7.21 – 7.12 (m, 2H), 7.04 (d, J = 7.9 Hz, 1H), 6.99 (t, J = 7.4 Hz, 1H), 5.31 (s, 2H). ^{13}C NMR (100 MHz, DMSO) δ 144.58, 144.33, 143.30, 135.23, 132.93, 129.83, 128.41, 128.22, 127.97, 127.91, 127.31, 126.92, 123.36, 121.83, 121.66, 121.56, 119.20, 116.16, 112.03, 43.58. HR-MS (ESI): Calcd. $C_{22}H_{15}ClF_3N_4S$, $[M+H]^+$m/z: 459.0658, found: 459.0662.

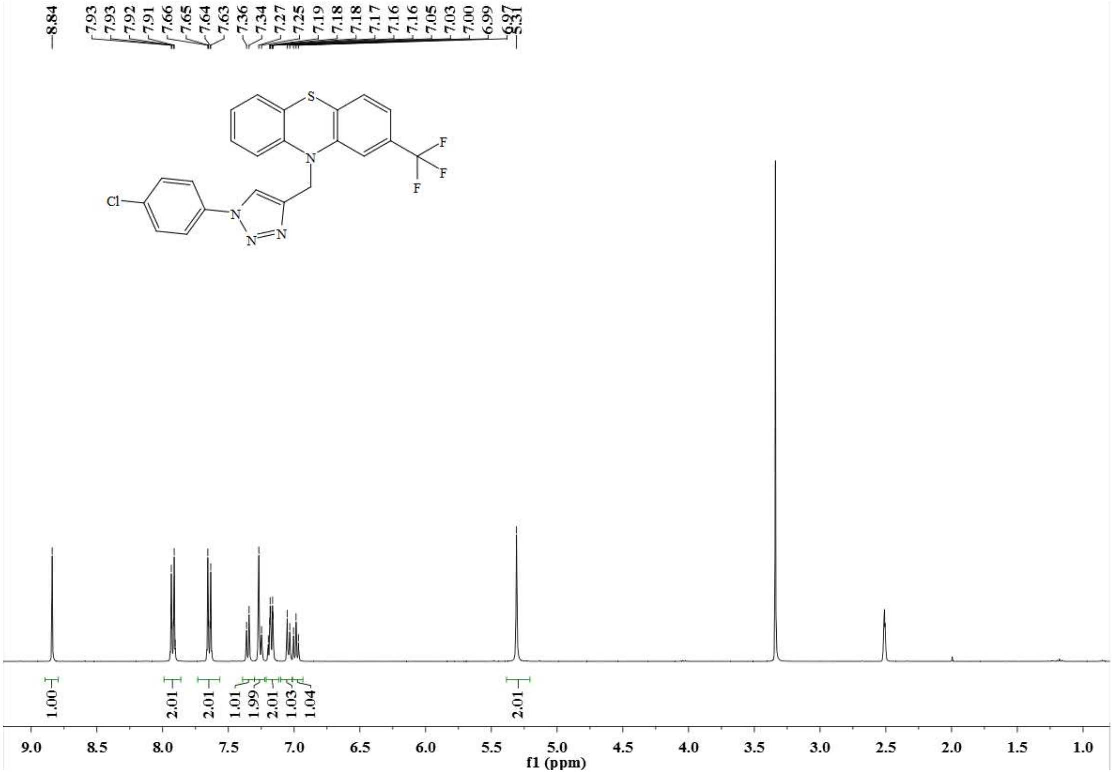

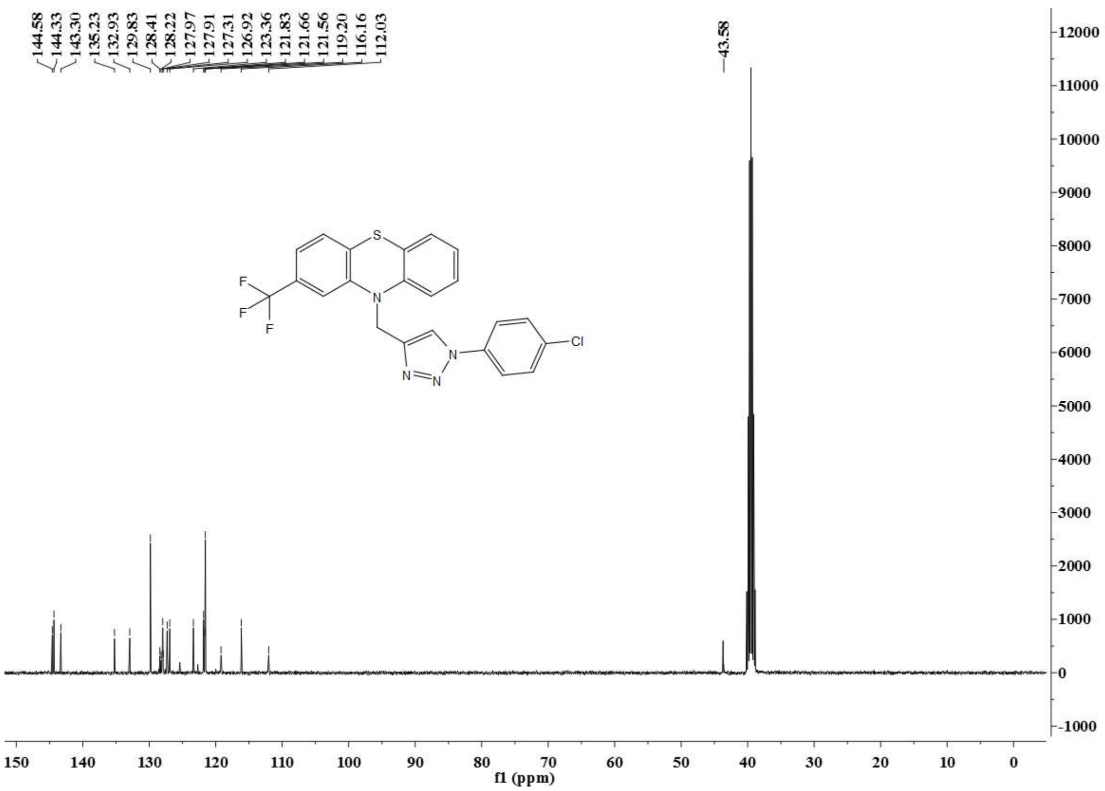

10-((1-(4-Fluorobenzyl)-1H-1,2,3-triazol-4-yl)methyl)-2-(trifluoromethyl)-10H-phenothiazine (9i)

Yield: 81%. White solid. Mp: 134~136ºC. ^{1}H NMR (400 MHz, DMSO) δ 8.07 (s, 1H), 7.33 (d, J = 7.9 Hz, 1H), 7.28 – 7.19 (m, 3H), 7.19 – 7.07 (m, 5H), 7.06 – 6.90 (m, 2H), 5.56 (s, 2H), 5.21 (s, 2H). ^{13}C NMR (100 MHz, DMSO) δ 144.44, 143.37, 143.27, 132.41, 132.38, 129.75, 129.66, 127.92, 127.30, 126.90, 125.35, 123.68, 123.31, 121.53, 119.16, 116.11, 115.49, 115.28, 111.93, 51.89, 43.88. HR-MS (ESI): Calcd. $C_{23}H_{17}F_4N_4S$, $[M+H]^+$m/z: 457.1110, found: 457.1118.

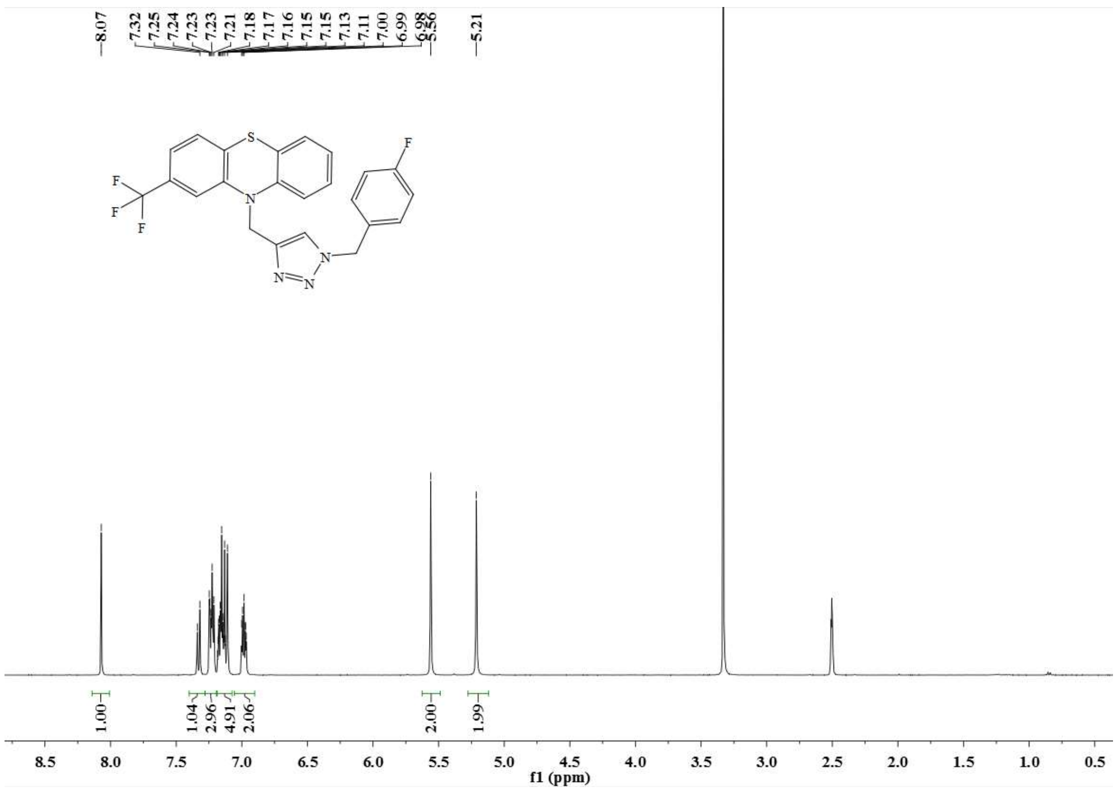

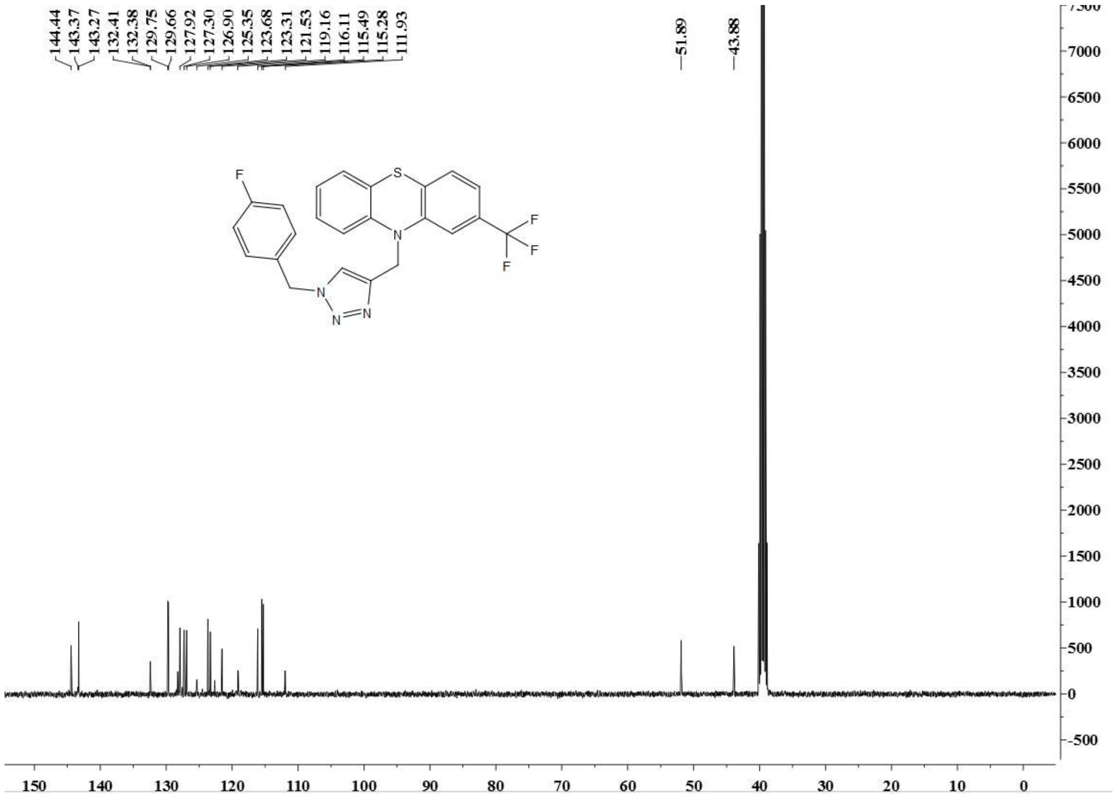

10-((1-Benzyl-1H-1,2,3-triazol-4-yl)methyl)-2-(trifluoromethyl)-10H-phenothiazine (9j)

Yield: 79%. White solid. Mp: 138~139ºC. ^{1}H NMR (400 MHz, DMSO) δ 8.07 (s, 1H), 7.39 – 7.27 (m, 4H), 7.24 (d, *J* = 8.1 Hz, 1H), 7.20 – 7.07 (m, 5H), 7.03 – 6.93 (m, 2H), 5.58 (s, 2H), 5.22 (s, 2H). ^{13}C NMR (100 MHz, DMSO) δ 144.45, 143.38, 143.21, 136.19, 128.56, 128.24, 128.18, 127.90, 127.30, 127.24, 126.89, 125.37,

123.82, 123.31, 122.66, 121.51, 119.08, 116.12, 111.97, 52.62, 43.92. HR-MS (ESI): Calcd. $C_{23}H_{18}F_3N_4S$, $[M+H]^+$m/z: 439.1204, found: 439.1208.

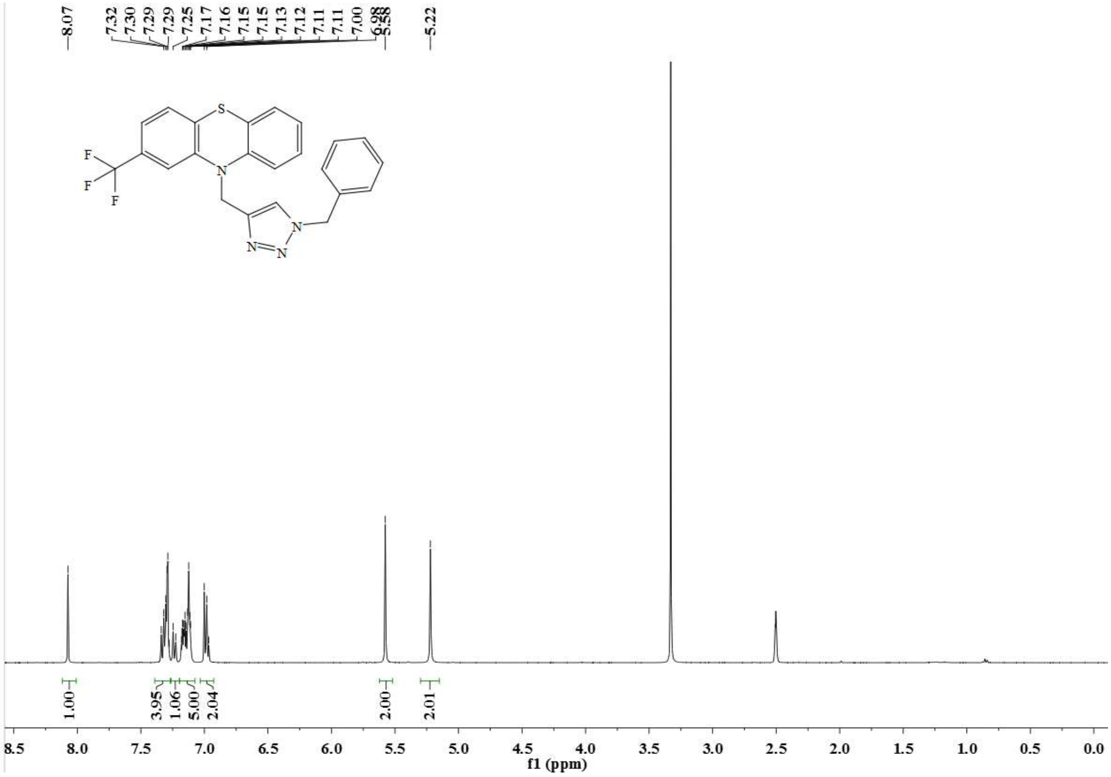

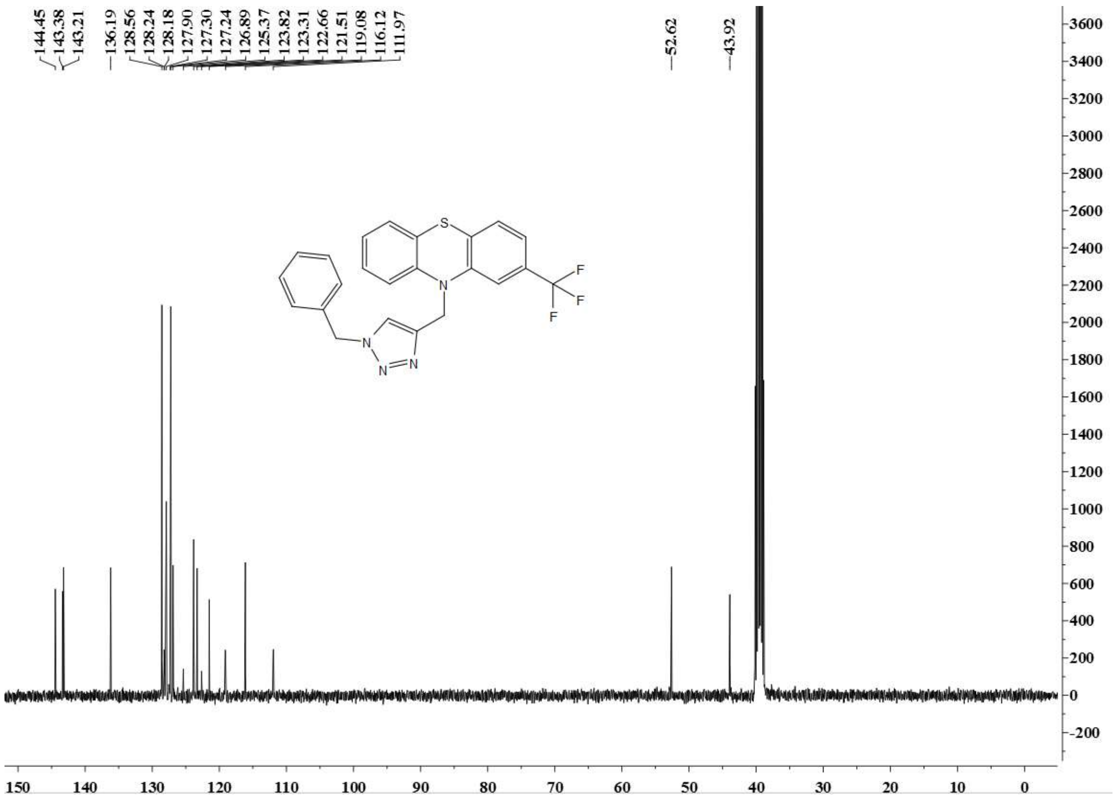

10-((1-(4-Chlorobenzyl)-1H-1,2,3-triazol-4-yl)methyl)-2-(trifluoromethyl)-10H-phenothiazine (9k)

Yield: 86%. White solid. Mp: 140~142°C. ^{1}H NMR (400 MHz, DMSO) δ 8.08 (s, 1H), 7.43 – 7.28 (m, 3H), 7.24 (d, J = 8.0 Hz, 1H), 7.17 (ddd, J = 5.7, 3.8, 2.4 Hz, 4H),

7.12 (s, 1H), 7.05 – 6.85 (m, 2H), 5.58 (s, 2H), 5.22 (s, 2H). ^{13}C NMR (100 MHz, DMSO) δ 144.44, 143.31, 135.17, 132.66, 129.31, 128.54, 128.27, 128.17, 127.92, 127.85, 127.31, 126.90, 123.82, 123.31, 121.55, 119.13, 119.09. 116.12, 111.89, 51.88, 43.89. HR-MS (ESI): Calcd. $C_{23}H_{17}ClF_3N_4S$, $[M+H]^+$m/z: 473.0815, found: 473.0819.

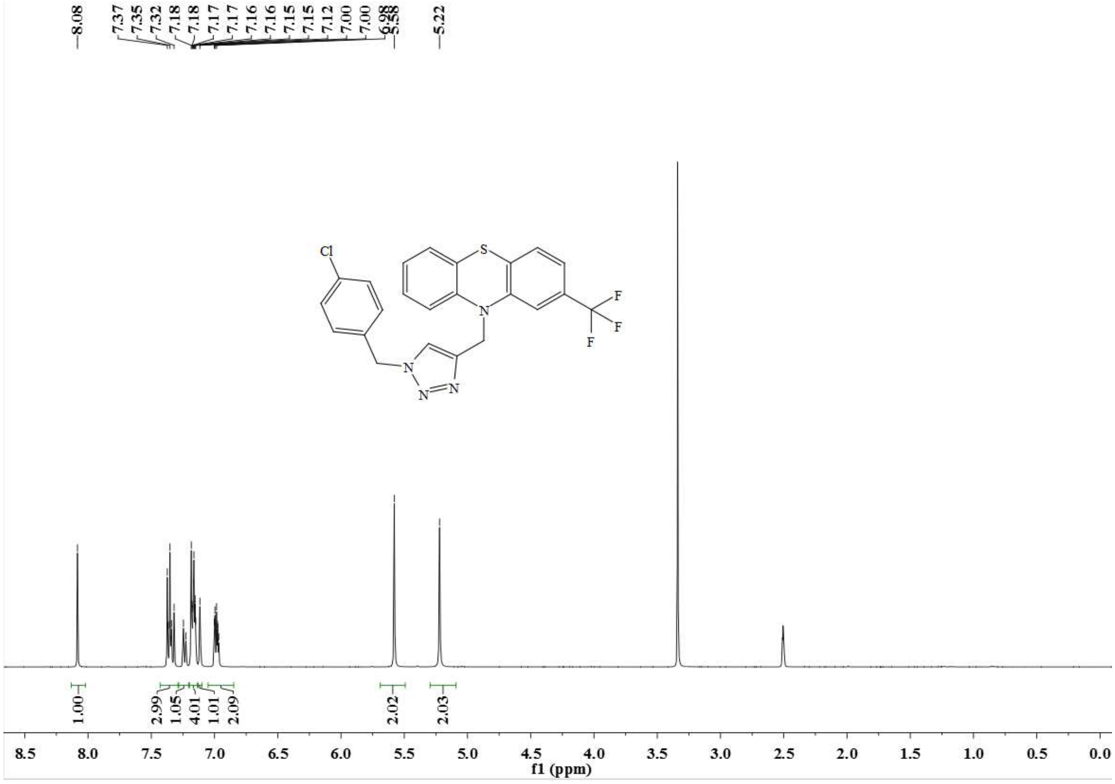

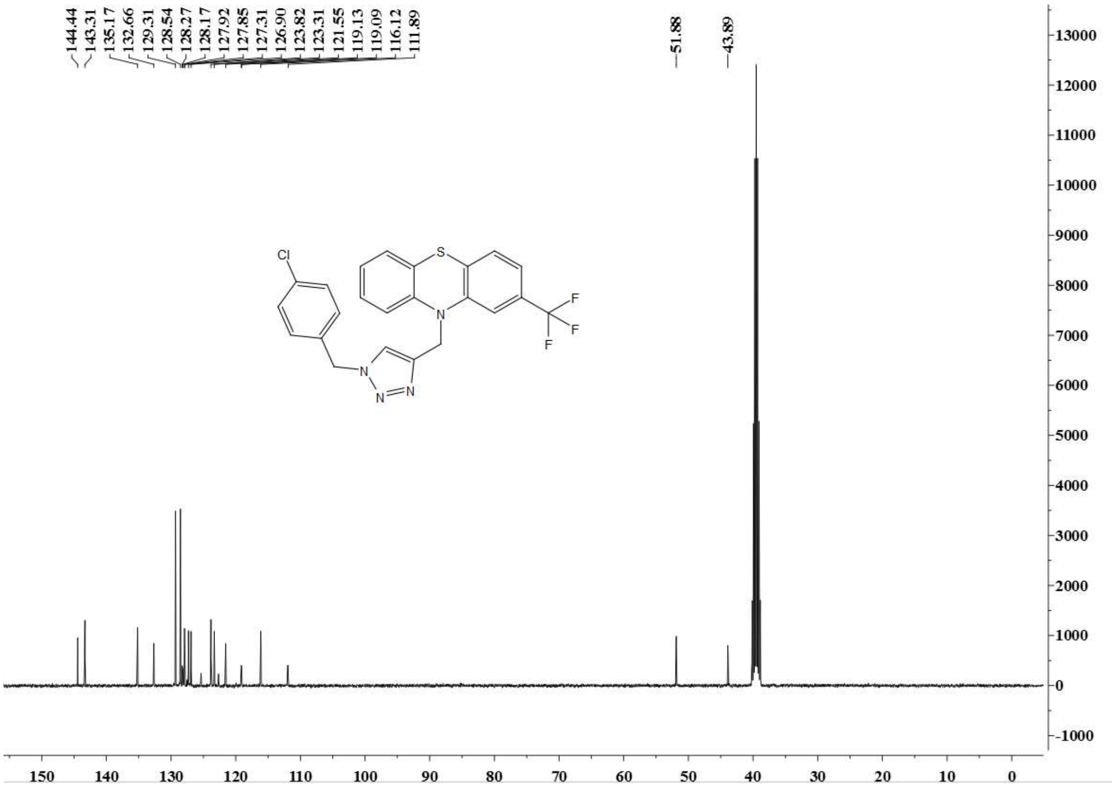

Supplement: Supplementary file 1 [file molecules-23-01288-s001.pdf]
